# Evolutionary insights into *Cordyceps militaris* from mitogenome dynamics and genome-wide variation

**DOI:** 10.3897/imafungus.17.195879

**Published:** 2026-08-12

**Authors:** Li-Yuan Ren, Shu Zhang, Yong-Jie Zhang

**Affiliations:** 1 School of Life Science, Shanxi University, Taiyuan 030031, China School of Life Science, Shanxi University Taiyuan China; 2 Key Laboratory of Chemical Biology and Molecular Engineering of Ministry of Education, Shanxi University, Taiyuan 030006, China Biomedicine and Health Laboratory in Shanxi Province, Shanxi University Taiyuan China; 3 Biomedicine and Health Laboratory in Shanxi Province, Shanxi University, Taiyuan 030031, China Key Laboratory of Chemical Biology and Molecular Engineering of Ministry of Education, Shanxi University Taiyuan China

**Keywords:** *Cordyceps militaris*, evolution, intron, mitogenome, nuclear DNA, phylogeny

## Abstract

*Cordyceps militaris* is a prized entomopathogenic fungus with significant medicinal and commercial value, yet its intraspecific genetic diversity has not been thoroughly examined. Mitogenomes from 118 geographically diverse samples were analyzed to trace the evolutionary trajectory of *C. militaris* and elucidate the diversification of its mitochondrial genome (mitogenome). These mitogenomes are circular DNA molecules with lengths ranging from 26.5 to 40.3 kbp and GC contents ranging from 26.12% to 27.24%. Comparative mitogenomic analyses revealed conserved gene content and syntenic relationships among various samples, with intraspecific divergence primarily driven by dynamic intron turnover (including 18 identified insertion sites, with deletions outnumbering acquisitions) and high variability in intergenic regions. Evidence of mitochondrial-to-nuclear DNA transfer was also detected. Notably, the mitogenome evolved at an accelerated rate, accumulating mutations approximately 2.8-fold faster than the nuclear genome (SNP frequencies: 1.58% vs. 0.57%). Phylogenomic reconstructions based on mitochondrial and nuclear genomes yielded largely congruent topologies but with subtle discordances, reflecting the complex coevolutionary relationship between the two genomic compartments. Collectively, this study provides a comprehensive view of mitogenome architecture and evolutionary dynamics in *C. militaris*, reveals substantial organellar genetic diversity, and underscores the critical role of mitogenomes in understanding organelle–nuclear coevolution.

## Introduction

*Cordyceps* is one of the most species-rich genera of entomopathogenic fungi within the family *Cordycipitaceae* (*Hypocreales*, *Sordariomycetes*, *Ascomycota*), comprising approximately 254 accepted species (https://nmdc.cn/fungalnames/; accessed 8 June 2026). Species of *Cordyceps* are pathogenic to a variety of insect pests, acting as natural biocontrol agents that regulate insect populations and maintain ecosystem functioning (Evans 1982). Among them, *Cordyceps militaris* (L.) Fr. is a medicinally and economically significant entomopathogenic fungus and serves as a model organism for studying the genus (Sung et al. 2007). The species completes its life cycle through parasitism mainly on lepidopteran larvae and pupae in the humus-rich topsoil layer of temperate broadleaf and mixed forest ecosystems (Zhang et al. 2015). Extensive chemical analyses have identified diverse bioactive metabolites in *C. militaris*, including cordycepin (Tuli et al. 2013), pentostatin (Xia et al. 2017), immunomodulatory proteins (Yang et al. 2015), novel water-soluble carotenoids (Dong et al. 2013), and *N*^6_^(2-hydroxyethyl)-adenosine (Wu et al. 2021). These compounds confer broad-spectrum pharmacological activities, such as antibacterial (Lee et al. 2015), antitumor (Wu et al. 2007), antioxidant (Kim et al. 2006), anti-inflammatory (Noh et al. 2009), and immunostimulatory effects (Bi et al. 2018), highlighting their dual value in therapeutic and functional food applications. The successful artificial cultivation of its fruiting bodies has led to regulatory approval of *C. militaris* as a novel food in China (Dong et al. 2015). Multi-omics studies have since elucidated its genomic (Zheng et al. 2011), mitogenomic (Zhang et al. 2015), epigenomic (methylome) (Chen et al. 2019), transcriptomic (Chen et al. 2019), proteomic (Yin et al. 2012), and metabolomic (Vongsangnak et al. 2017) landscapes. Nevertheless, significant gaps persist in understanding its intraspecific genetic variation, thereby limiting systematic assessments of its genetic diversity and adaptive evolution.

Genetic diversity serves as a fundamental driver of species adaptation in changing environments. Modern molecular genetics leverages mitochondrial, ribosomal, and microsatellite markers to analyze haplotypes, reconstruct phylogenies, and assess polymorphism patterns (Menz et al. 2004). Among these, mitochondrial genomes (mitogenomes) represent a vital “second genome” in eukaryotes (Jorgensen et al. 2018), possessing several useful features, including compact size, high copy number, maternal inheritance, low recombination rates, conserved core gene composition, and relatively rapid evolution at the nucleotide sequence level (Paquin et al. 1997; Kouvelis et al. 2004; Joardar et al. 2012). These features make them particularly useful for reconstructing phylogenetic relationships and tracing evolutionary trajectories independent of nuclear genomic influences (Xu and Wang 2015). Fungal mitogenomes are typically circular double-stranded DNA molecules, and both their sizes and size ranges are smaller than those of nuclear genomes but intermediate between those of animal and plant mitogenomes (Tang et al. 2024). The sizes of fungal mitogenomes exhibit considerable variability, with smaller ones, such as that of *Hanseniaspora uvarum*, measuring only 11 kb (Pramateftaki et al. 2006), and larger ones, such as that of *Morchella crassipes*, reaching 531.2 kb (Liu et al. 2020). Since the first *C. militaris* mitogenome was sequenced (Sung 2015), subsequent studies have characterized its mitochondrial diversity, revealing intron polymorphisms as a primary driver of intraspecific differentiation (Zhang et al. 2015). An analysis of 11 complete mitogenomes further identified region-specific diversity, with only a minimal contribution from recombination (Zhang et al. 2017a). However, systematic comparisons of mitogenome divergence across a broad geographic range remain lacking.

Nuclear DNA (nuDNA) ensures genomic stability through stringent chromatin regulation and robust DNA repair mechanisms, while mitochondrial DNA (mtDNA) frequently exhibits divergent evolutionary trajectories across different lineages (Serrano et al. 2024). Comparative genomic analyses reveal striking phylogenetic patterns in their evolutionary rates. In plants and most fungi, mtDNA generally evolves at a significantly slower rate than nuDNA, whereas the opposite trend predominates in animals. Specifically, variation in bilaterian mtDNA exceeds that in nuDNA by 9- to 25-fold, largely attributable to oxidative damage and reduced repair efficiency, while plant mtDNA evolves approximately 20 times more slowly, reflecting stringent selective constraints on respiratory genes (Palmer and Herbon 1989; Lynch et al. 2006; Sandor et al. 2018). Fungal systems demonstrate particularly complex variation. For instance, *Saccharomyces cerevisiae* displays ~37-fold greater mtDNA variation (Lynch et al. 2008), whereas *Lachancea thermotolerans* (nuDNA polymorphism index = 0.012 vs. mtDNA = 0.0014) (Freel et al. 2014) and *Tolypocladium inflatum* (nuclear SNP frequency = 0.97% vs. mtDNA = 0.12%) (Zhang et al. 2017b) exhibit faster nuDNA evolution. Previous studies have shown that the distinct biological characteristics of mitochondrial and nuclear genomes can result in discordant evolutionary histories, even though they coexist within the same cell (De Chiara et al. 2020). In *C. militaris*, a previous analysis based on 14 mtDNA and seven nuDNA loci identified moderately accelerated nuDNA variation (SNP frequencies: 0.71% vs. 1.24%, a 1.7-fold difference), generating phylogenetically discordant topologies (Zhang et al. 2019). However, locus-specific comparisons may not fully capture genome-wide dynamics, highlighting the necessity of integrated whole-genome analyses to resolve mitochondrial–nuclear evolutionary disparities and their functional and phylogenetic implications.

The primary objective of this study was to systematically characterize the evolutionary dynamics of the *C. militaris* mitogenome. To this end, complete mitogenomes from 118 geographically diverse samples were assembled and compared to resolve their architectural features, haplotype diversity, syntenic relationships, intron polymorphism and turnover dynamics, nucleotide variations, and phylogenetic relationships. For a complementary nuclear perspective, 90 nuclear genomes from *C. militaris* strains were further screened for potential mtDNA insertions, and genome-wide SNP genotyping and phylogenomic reconstruction were performed. Specifically, this study aimed to (1) comprehensively characterize the intraspecific variation of the *C. militaris* mitogenome and (2) compare the evolutionary rates and patterns between the mitochondrial and nuclear genomes. This integrated comparative analysis provides comprehensive genomic resources and new insights into organelle–nuclear coevolution in this economically important medicinal fungus.

## Materials and methods

### Fungal samples used in this study

**Figure 1.**
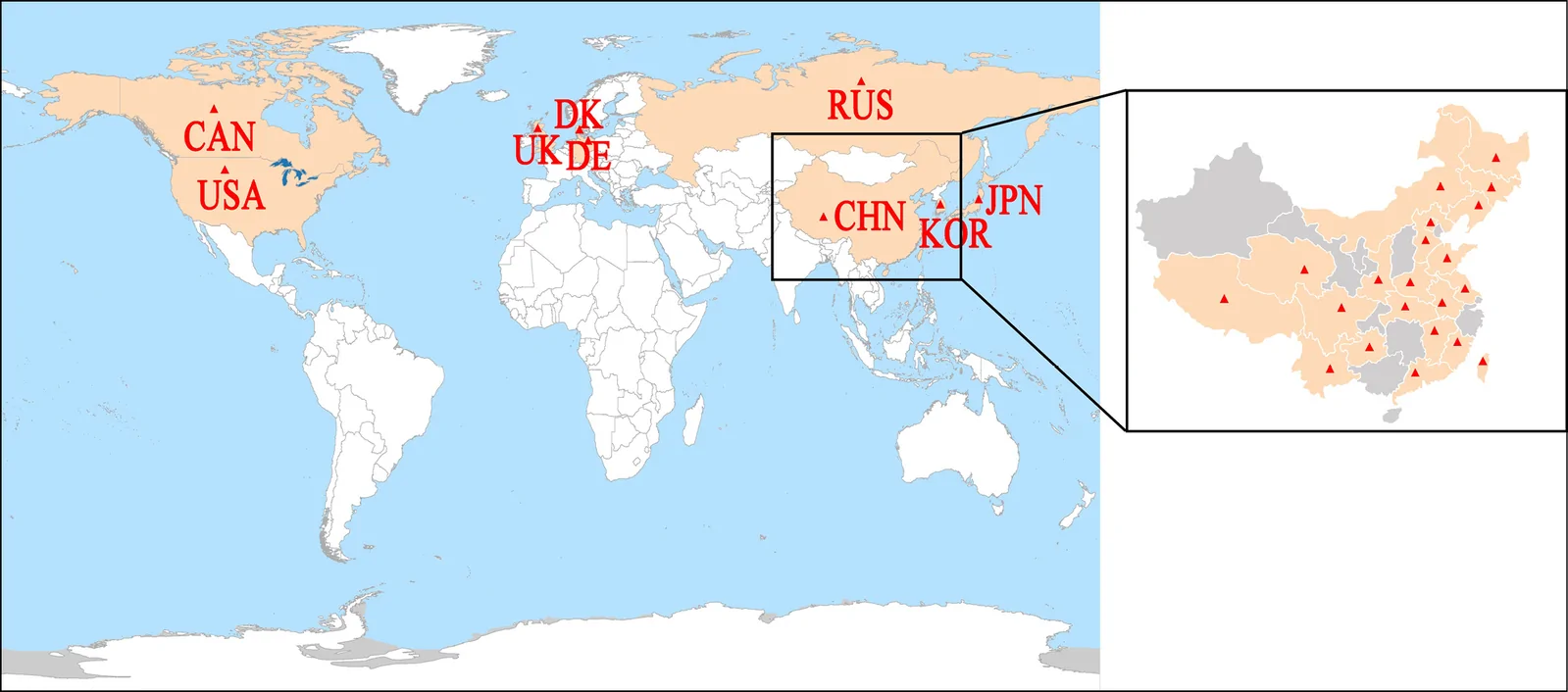
Sampling locations of *C. militaris* samples. This map shows the geographic origins of 93 samples with original sampling locations (red triangles), whereas 25 samples with unknown origins are omitted. As multiple samples share identical collection localities, only the nine countries and 21 Chinese provinces represented in the dataset are shown. Corresponding locality details are listed in Suppl. material 1: table S1.

This study incorporated 118 *C. militaris* samples obtained through collaborations with major culture collection centers (including CGMCC, ACCC, CFCC, and RCEF, among others) and academic research institutions (Suppl. material 1: table S1). The sample set consisted of three categories: (1) 89 laboratory-maintained or commercially developed strains (designated with the prefix ZYJ); (2) 26 wild or cultivated fruiting bodies (prefix FSXU); and (3) three samples sourced from public databases, namely HN001 (with both mitochondrial and nuclear genome assemblies available), EFCC-C2 (with mitogenome data only), and DMS-9329726 (NCBI SRA accession: SRR12518718). Of these, 93 samples had confirmed origins (i.e., original sampling locations), including 74 strains and 19 fruiting bodies, representing nine countries across North America (CAN and USA), Europe (DK, UK, DE, and RUS), and East Asia (CHN, JPN, and KOR) (Fig. 1). Samples from China were overrepresented, with 80 originating from 21 provinces. The remaining 25 samples lacked geographic data; 13 of these were company-sourced strains or fruiting bodies (Suppl. material 1: table S1).

Genomic DNA was extracted from all *C. militaris* samples for subsequent analyses. The strains were first grown on potato dextrose agar overlaid with sterilized cellophane and incubated at 25 °C for 10 days. After the mycelia or fruiting bodies were harvested, total DNA was isolated using an optimized cetyltrimethylammonium bromide (CTAB) protocol (Zhang et al. 2010), ensuring high-molecular-weight DNA suitable for sequencing.

To taxonomically validate all samples, a multilocus approach targeting six nuclear regions was employed: the ribosomal DNA cluster (ITS, LSU, and SSU) and three protein-coding genes (*tef1α*, *rpb1*, and *rpb2*). Sequences for these loci were identified in the genome assembly of each sample (see the methods described below) via local BLAST searches, using the following sequences as queries: ITS (GenBank accession JN049825), LSU (AY184966), SSU (AY184977), *tef1α* (DQ522332), *rpb1* (DQ522377), and *rpb2* (AY545732). Phylogenetic analysis of the concatenated sequences, using the reference fungal taxa detailed in Suppl. material 1: table S2, confirmed that all samples belonged to *C. militaris* (Suppl. material 2: fig. S1).

### Genome sequencing, assembly, and annotation

Genomic DNA extracted from both harvested mycelia and fruiting bodies was sheared into ~350 bp fragments for the construction of high-throughput sequencing libraries. Sequencing was performed on an Illumina NovaSeq 6000 platform (2 × 150 bp; Novogene Co., Ltd., Tianjin, China). Raw sequencing data were filtered by removing adapter sequences, low-quality bases (*Q* < 20), and reads containing excessive ambiguous nucleotides (“N”). The resulting high-quality clean reads were retained for downstream analyses. Nuclear genomes were assembled using IDBA v1.1.3 (Peng et al. 2010), and mitogenomes were assembled using two programs: NOVOPlasty v4.3.1 (Dierckxsens et al. 2017) and GetOrganelle v1.7.5 (Jin et al. 2020). Mitogenome completeness and circularity were evaluated by integrating the circular assembly graph generated by GetOrganelle, independent circular assemblies produced by NOVOPlasty, and comparisons with the corresponding whole-genome assemblies. Where gaps remained in the initial mitochondrial outputs, local BLAST searches against the published *C. militaris* reference mitogenome (GenBank accession no. KP722501) were performed to precisely identify the missing regions. Subsequently, specific PCR primer pairs were designed from the known flanking regions to amplify the putative gap regions. PCR amplification was carried out using high-fidelity KOD FX Neo DNA polymerase (TOYOBO Bio-Technology Co., Osaka, Japan), and the resulting amplicons were validated through Sanger sequencing (GENEWIZ Inc., Suzhou, China).

Complete mitogenome sequences were functionally annotated according to established methods (Zhang et al. 2015). For precise intron characterization, sequence alignments of intron-containing genes against intron-free reference genes were performed. Mitochondrial introns were named according to standardized intron nomenclature (Zhang and Zhang 2019). Intron types were predicted using the online tool RNAweasel (https://megasun.bch.umontreal.ca/apps/rnaweasel/).

### Comparison among different *C. militaris* mitogenomes

To investigate mitogenome evolution in *C. militaris*, a comprehensive comparative analysis of base composition, mitogenome size, gene order, intron distribution, genetic divergence, selection pressure, and codon usage bias was conducted. Nucleotide composition biases were quantified through strand asymmetry analysis, calculated as AT-skew = [*A* − *T*]/[*A* + *T*] and GC-skew = [*G* − *C*]/[*G* + *C*]. Mitogenome sequences were aligned using MAFFT v7.471 (Rozewicki et al. 2019) to evaluate structural and sequence-level differences. To clarify genetic differentiation among *C. militaris* samples, mitochondrial haplotype (hereafter “mitotype”) diversity was identified using DnaSP v6.10.01 (Rozas et al. 2003) based on complete mitogenome sequences. A haplotype network based on the concatenated nucleotide sequences of the 14 conserved mitochondrial protein-coding genes (PCGs) was then constructed using POPART v1.7 (Leigh and Bryant 2015). Building on the mitotype data, a mitogenome-wide collinearity analysis using Mauve v2.3.1 (Darling et al. 2010) was conducted to assess structural conservation and identify potential rearrangement events across the identified mitotypes.

To further explore the evolutionary dynamics of mitochondrial genes, a comprehensive comparative analysis was conducted. Structural variations were cataloged through systematic comparisons of intron insertion sites, with their molecular organization graphically represented using IBS v1.0.3 (Liu et al. 2015). A pan-intronic landscape was subsequently generated and visualized using Circos v0.69-9 (Krzywinski et al. 2009), thereby providing a holistic view of intronic diversity patterns across all identified mitotypes. Base compositional analysis of these mitogenomes was conducted using MEGA v11.0.13 (Tamura et al. 2021), complemented by a detailed examination of 14 PCGs, with a specific focus on their amino acid profiles and codon usage patterns under genetic code 4. Evolutionary dynamics were further evaluated using DnaSP v6.10.01 (Rozas et al. 2003), which enabled the calculation of synonymous (*K*s) and nonsynonymous (*K*a) substitution rates for the 15 PCGs (14 conserved PCGs plus *rps3*), whereas pairwise genetic distances across mitotypes were estimated using the Kimura two-parameter (K2P) model in MEGA v11.0.13 (Tamura et al. 2021).

### Origin and evolution of mitochondrial introns

To investigate the possible origin of mitochondrial introns identified in *C. militaris* mitogenomes, a comparative genomic approach was employed. First, pairwise alignments of intronic sequences, along with their encoded homing endonuclease protein sequences, were performed using the EMBOSS Stretcher tool (https://www.ebi.ac.uk/jdispatcher/psa/emboss_stretcher) to detect potential duplication events. Subsequently, online BLASTN searches were conducted for each intron to assess the possibility of horizontal gene transfer. To ensure rigor, stringent filtering criteria were applied: only matches with ≥ 80% sequence identity and ≥ 90% query coverage were retained, thereby ensuring high-confidence homology detection.

To characterize mitochondrial intron distribution patterns, the binary presence (1) or absence (0) of 18 intron sites across 71 *C. militaris* mitotypes (defined below) was recorded, and profile similarities were analyzed by constructing a dendrogram using the online tool DendroUPGMA (http://genomes.urv.cat/UPGMA/). To reconstruct the evolutionary history of introns, a phylogenetic analysis was conducted based on concatenated exon sequences from 14 PCGs using the optimal substitution model determined by PartitionFinder v2.1.1 (Lanfear et al. 2017). Phylogenetic trees were reconstructed using MrBayes v3.2.7a for Bayesian inference (BI) (Ronquist et al. 2012) and RAxML v8.2.12 for maximum likelihood (ML) analysis (Stamatakis 2014), with *Cordyceps chanhua* designated as the outgroup (Suppl. material 1: table S3). The topology was then used to map intron presence (1) and absence (0) patterns, from which the minimum number of intron gain and loss events was inferred under a parsimony framework using Mesquite v4.0 (Maddison and Maddison 2025).

### Detection of NUMTs in nuclear genomes

To evaluate mitochondrial-to-nuclear DNA transfer, a curated dataset of 90 *C. militaris* strains (89 newly sequenced strains plus the public strain DMS-9329726) was compiled. Fruiting-body samples were omitted to avoid potential exogenous contamination. For each strain, the mitogenome was used as a query in a local BLAST search against its corresponding whole-genome assembly. Candidate nuclear mitochondrial DNA segments (NUMTs) were identified as sequences with an E-value < 10^–5^ and > 75% identity after filtering out contigs showing > 99.5% identity and coverage (i.e., those likely representing mitochondrial sequences). A representative subset of long NUMTs (>400 bp) was selected for experimental validation. For each candidate, primers were designed to flank the targeted NUMT locus, and the presence of the NUMT was confirmed by PCR amplification followed by agarose gel electrophoresis.

### Evolutionary comparison between mitochondrial and nuclear genomes

To estimate nuclear variation across the 90 *C. militaris* strains, the *C. militaris* strain CM01 (NCBI accession GCA_000225605) was used as the nuclear reference genome, with its mitochondrial fragments excluded. Clean sequencing reads from all strains were aligned to the reference genome using BWA-MEM v0.7.19 (Li 2013), followed by read sorting and PCR duplicate removal using Picard v2.25.6 (Picard Toolkit 2019). Variant calling was performed using SAMtools v1.22.1 (Li et al. 2009) and vcftools v0.1.16 (Danecek et al. 2011), with subsequent SNP and indel detection using GATK v3.5 (McKenna et al. 2010). Detected variants were annotated using ANNOVAR (Yang and Wang 2015), and their distribution across strains was statistically analyzed.

For phylogenetic inference, a nuclear SNP-based tree was constructed using the ML method in RAxML v8.2.12 (Stamatakis 2014), while the mitochondrial phylogeny was derived from whole-mitogenome alignments under the same ML framework. To assess topological concordance between the nuclear and mitochondrial evolutionary histories, tanglegrams were generated by comparing the two trees using TreeMap v3b (Charleston 2011). The statistical significance of topological differences between the mitochondrial and nuclear genome phylogenies was assessed using the Kishino–Hasegawa (KH) and Shimodaira–Hasegawa (SH) tests in PAUP v4.0b10 (Swofford 2002). Finally, results from NUMT identification, evolutionary rate comparisons, and phylogenetic analyses were integrated to assess mitochondrial–nuclear interactions and evolutionary concordance.

## Results

### Organization of *C. militaris* mitogenomes

Complete circular mitogenomes were obtained for all 118 *C. militaris* samples. These mitogenomes ranged in size from 26,527 bp (e.g., ZYJ0540 from Russia) to 40,362 bp (e.g., ZYJ1057 from China), with a mean length of 31,315 ± 3,152 bp (Suppl. material 1: table S1). They exhibited highly constrained GC contents (26.12–27.24%). Strand asymmetry analysis, calculated with the forward strand as the reference, revealed that AT-skew was positive in 61% of the samples and negative in the remaining 39%, indicating the absence of a consistent strand bias between adenine (A) and thymine (T). In contrast, GC-skew was uniformly positive (0.128–0.136) across all mitogenomes, indicating a stable bias toward guanine (G) over cytosine (C) on the forward strand.

All *C. militaris* mitogenomes contained a conserved suite of 14 PCGs essential for oxidative phosphorylation: seven NADH dehydrogenase subunits (*nad1–nad6*, *nad4L*; Complex I), one cytochrome *b* (*cob*; Complex III), three cytochrome *c* oxidase subunits (*cox1–cox3*; Complex IV), and three ATP synthase components (*atp6*, *atp8*, *atp9*; Complex V) (Suppl. material 1: table S4). These core genes were consistently accompanied by two rRNA genes (*rnl* and *rns*), a nearly complete set of tRNA genes, and, in some cases, multiple non-conserved open reading frames (ncORFs). These tRNA genes (*n* = 26–27) were distributed across three primary clusters: five upstream of *rnl* (*trnV*, *I*, *S*, *W*, *P*), 12 downstream of *rnl* (*trnT*, *E*, *M*, *M*, *L*, *A*, *F*, *K*, *L*, *Q*, *H*, *M*), and five downstream of *rns* (*trnY*, *D*, *S*, *N*, *R*). Intriguingly, 5% of these samples (6/118) exhibited a *trnG_1*(*acc*) deletion while retaining its counterpart *trnG_2*(*ucc*) at the *cox3*/*nad6* junction, a finding confirmed by targeted PCR validation (Suppl. material 2: fig. S2). The number of ncORFs per sample varied from zero to four, with a total of 10 unique ncORF loci identified among all samples (Suppl. material 1: table S4). Notably, the two largest mitogenomes—ZYJ1057 (40.3 kb) and ZYJ1074 (39.3 kb)—each harbored four ncORFs, suggesting a potential link between ncORF accumulation and mitogenome size expansion. These ncORFs encoded LAGLIDADG (1/10) or GIY-YIG (4/10) homing endonucleases along with uncharacterized proteins (5/10), highlighting the remarkable structural plasticity of *C. militaris* mitogenomes.

To assess the factors driving mitogenome size variation, the *C. militaris* mitogenome was partitioned into three distinct regions: exons (the transcribed sequences of 14 core PCGs, two rRNAs, all available tRNAs, and putative ncORFs), introns (non-coding sequences interrupting genes), and intergenic regions (spaces between annotated genes). Among these, exons constituted the largest proportion of the mitogenome (mean, 64.58%; range, 52.23–74.59%) and exhibited length conservation across samples (19,678–22,783 bp; Suppl. material 2: fig. S3). Introns, in contrast, exhibited substantial variability (3,020–14,327 bp, 11.26–37.96% of mitogenome length, mean 22.67%) and emerged as major drivers of mitogenome expansion, showing strong positive correlations between intron number/length and total mitogenome size (*R* = 0.948–0.969; Suppl. material 2: fig. S4A, B). While intergenic regions (length 3,530–4,667 bp, 9.46–15.00%, mean 12.75%) demonstrated a statistically significant correlation with mitogenome size, their biological impact was negligible (*R* = 0.049; Suppl. material 2: fig. S4C). This tripartite analysis highlights intron dynamics—rather than intergenic spacer accumulation or exon plasticity—as the dominant force shaping *C. militaris* mitogenome architecture.

### Mitotype and synteny analysis among the *C. militaris* mitogenomes

Comparative mitogenomic analysis of the 118 *C. militaris* samples revealed 71 distinct mitotypes, which exhibited markedly uneven distribution patterns. The most dominant mitotype (mitotype 1; 31,854 bp) represented 25.4% of all samples (30/118), encompassing 21 laboratory-maintained strains, two commercial strains, six commercial fruiting bodies, and one publicly available genome (Suppl. material 1: table S1). The second most prevalent mitotype (mitotype 2; 26,535 bp) accounted for 7.6% of the samples (9/118), occurring exclusively in laboratory-maintained strains—six with documented geographic origins and three with unknown origins. Notably, while 12.7% of the mitotypes formed minor lineages comprising 2–3 samples each (9/71, mitotypes 3–11), the vast majority appeared as unique singletons restricted to individual samples (84.5%, 60/71, mitotypes 12–71).

**Figure 2.**
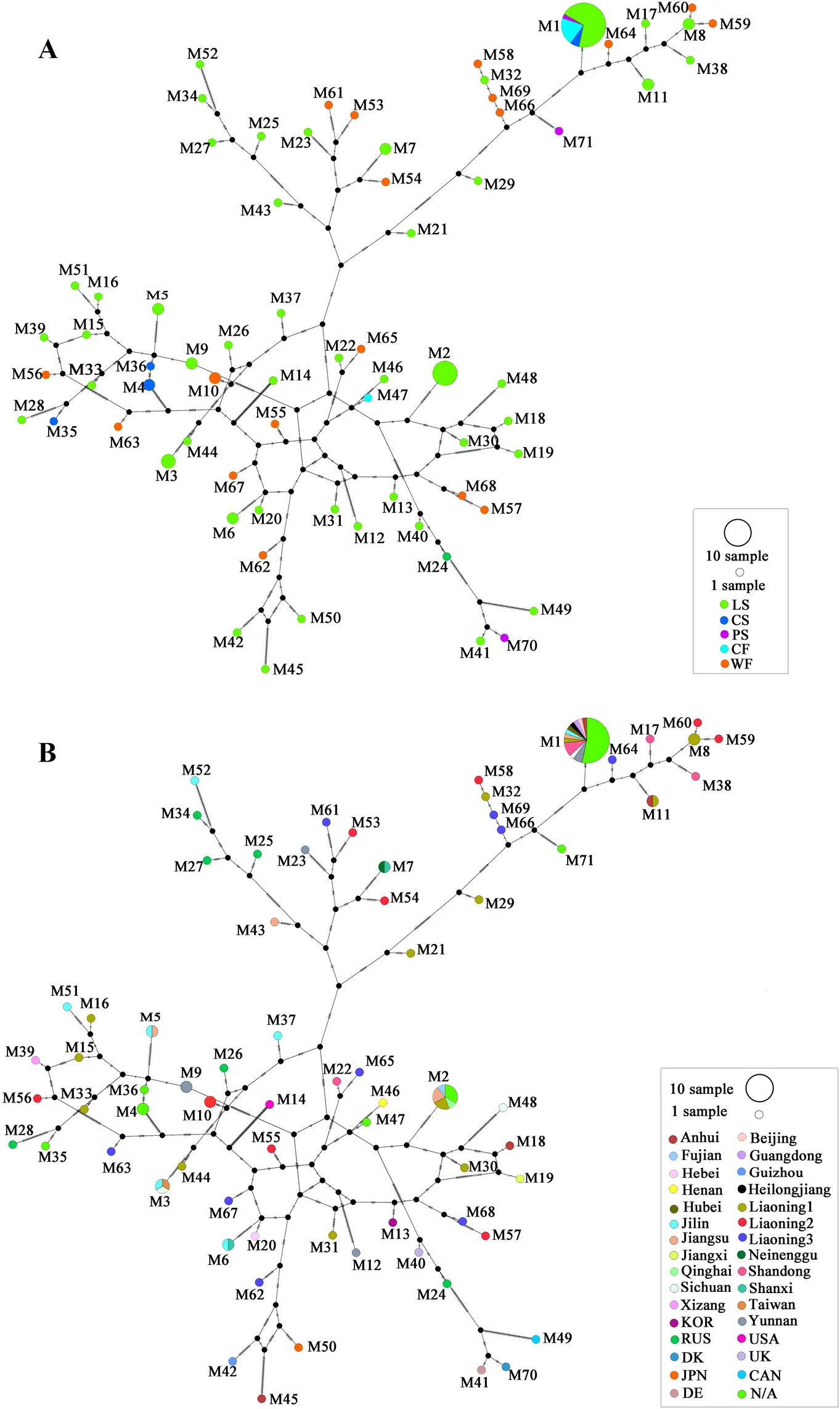
Haplotype networks of the 71 *C. militaris* mitotypes. **A**. Haplotype network colored by sample type: laboratory-maintained strains (LS), commercial strains (CS), public database strains (PS), wild fruiting bodies (WF), and commercial fruiting bodies (CF). **B**. Haplotype network colored by geographic origin. Samples from China are indicated by their province of origin, whereas samples from other countries are labeled by country name. “N/A” indicates that the geographic origin is unavailable or not documented. Samples from Liaoning Province in China were further subdivided into three subgroups: Liaoning1 (laboratory-maintained strains and commercial fruiting bodies), Liaoning2 (wild fruiting bodies from Tieling; mitotypes M10 and M53–60), and Liaoning3 (wild fruiting bodies from Fushun; mitotypes M61–69). Circle size is proportional to mitotype frequency. Tick marks along the connecting lines indicate inferred mutational steps, with each tick representing a single nucleotide substitution. Detailed sample locality information is provided in Suppl. material 1: table S1.

The mitotype network, reconstructed from 14 core PCGs and characterized by a radial stellate structure (Fig. 2A), revealed no discernible association between mitotype clustering and geographic origin. Mitotypes from different countries were widely dispersed across the network and did not form geographically distinct clusters, indicating a general lack of geographic population structure (Fig. 2B). Laboratory-maintained strains (*n* = 83) constituted the majority of the mitotypes (47 in total) and formed the central framework of the network. Commercial strains (*n* = 6) comprised four distinct mitotypes (1, 4, 35, and 36), exhibiting substantial genetic diversity. Wild fruiting bodies (*n* = 19) from two locations (Tieling and Fushun) in Liaoning, China, each displayed high mitotype diversity (nine mitotypes per site) and were diffusely distributed across the network, suggesting notable genetic differentiation without strong geographic structuring (Fig. 2B). In contrast, commercial fruiting bodies (*n* = 7) contained only two mitotypes (1 and 47), demonstrating severe genetic bottlenecks and directional selection during artificial breeding and mass cultivation. When all commercial samples (*n* = 13, including both strains and fruiting bodies) were considered, five mitotypes were identified, corroborating the multiple origins of the developed lineages.

Comparative alignments of mitogenomes performed with Mauve revealed a high degree of structural synteny conservation, identifying nine core locally collinear blocks (LCBs A–I) that were consistently preserved across all samples (Suppl. material 2: fig. S5). Among these, LCB “C” was the largest conserved segment, encompassing the genes *rnl*, *nad2*, *nad3*, and *atp9*, whereas LCB “G,” the second-largest block, contained the genes *nad1*, *nad4*, *atp8*, *atp6*, *rns*, and *cox3*. In addition, two accessory LCBs (“J” and “K”) were found exclusively as a result of the insertion of a *cox3* intron (c*ox3*P219) in certain lineages. Collectively, these findings underscore the remarkable evolutionary stability of the *C. militaris* mitogenomes, as evidenced by the absence of frequent rearrangements, a pattern indicative of strong functional constraints on mitogenome organization.

### Intron dynamics among *C. militaris* mitotypes

**Figure 3.**
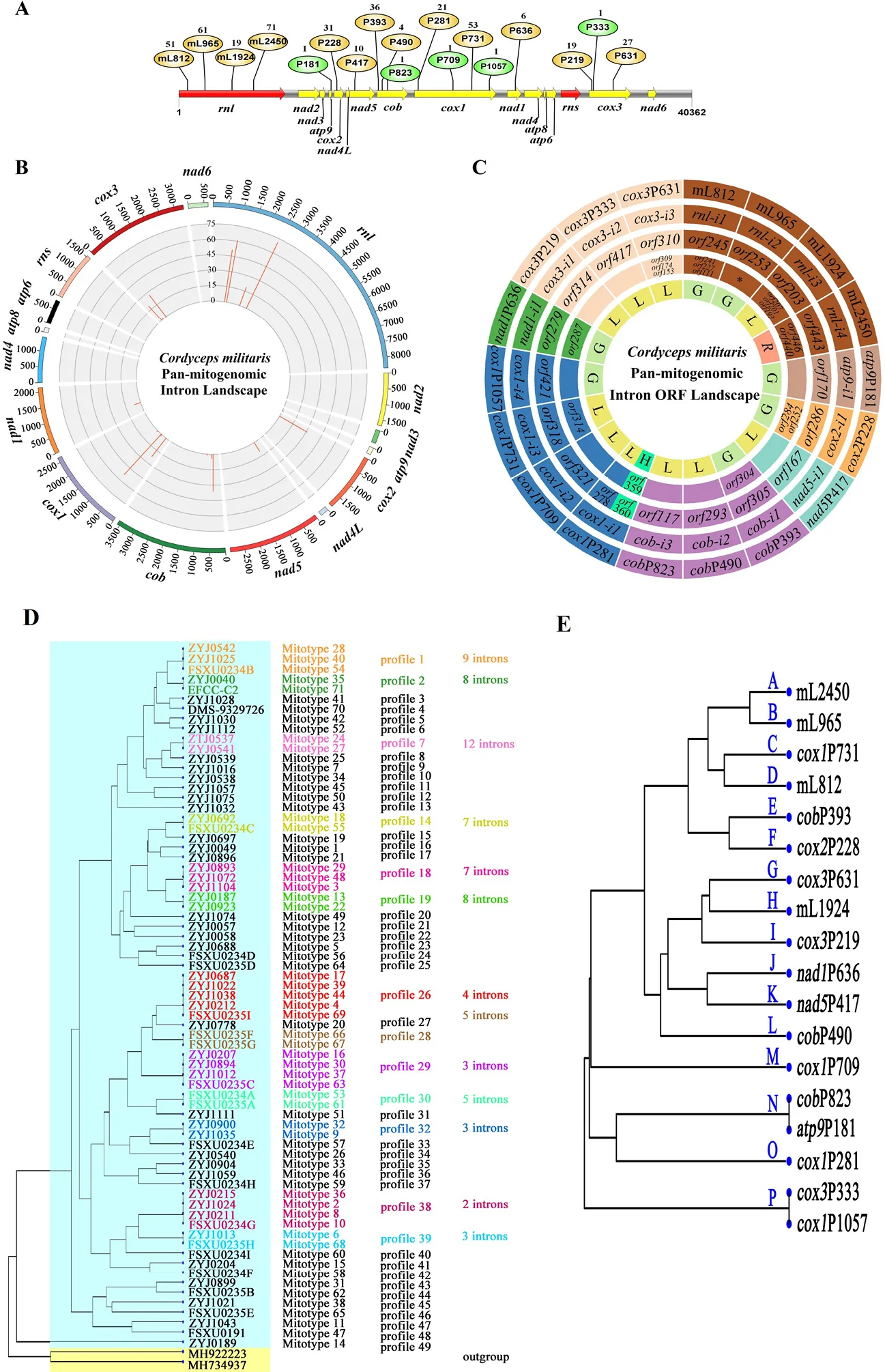
Distribution and characteristics of intron insertion sites and clustering analyses based on intron presence–absence data across 71 *C. militaris* mitotypes. **A**. Schematic map of intron insertion positions within eight protein-coding and rRNA genes, scaled to exon length. Numbers above the sites indicate the number of mitotypes containing each intron, all of which are group I introns. Site colors denote prevalence: green (unique to one mitotype) and yellow (shared by multiple mitotypes). **B**. Pan-intron profile showing the distribution of each insertion site across all mitotypes, where the height of the bars corresponds to the frequency of occurrence. **C**. Circular representation of intron-encoded ORF content and function. Rings from outer to inner display intron standard nomenclature, intron serial ID, common ORFs (shared by ≥ 2 introns), unique ORFs, and functional categories of IEPs (L: LAGLIDADG; G: GIY-YIG; H: hypothetical; R: Rps3). Asterisks (*) indicate ORFs with length polymorphism among mitotypes. **D**. Dendrogram of the 71 mitotypes clustered by their intron composition profiles across 18 intron loci. The 71 mitotypes yielded 49 distinct profiles: 36 were unique to a single mitotype (black labels), and 13 were shared by 2–4 mitotypes (colored labels). Mitotypes with identical total intron counts can possess different profiles. **E**. Dendrogram of the 18 intron loci clustered by their distribution patterns across all mitotypes. Sixteen distinct patterns were identified. Fourteen loci each displayed a unique pattern (A–M and O), while two pairs shared identical patterns: *cob*P823 and *atp9*P181 (pattern N), and *cox3*P333 and *cox1*P1057 (pattern P); each pair was exclusive to a single strain (ZYJ1074 and ZYJ0189, respectively).

A total of 18 distinct intron insertion sites (2–12 per sample) were identified across *C. militaris* mitogenomes, located within eight protein-coding and rRNA genes: *atp9*, *cob*, *cox1*, *cox2*, *cox3*, *nad1*, *nad5*, and *rnl* (Fig. 3A, B). The *rnl* and *cox1* genes contained the highest number of insertion sites (four each), followed by *cob* and *cox3* with three sites each, whereas the remaining genes (*atp9*, *cox2*, *nad1*, and *nad5*) each contained only a single intron. All introns belonged to the group I family and were classified into six subtypes: IB (*n* = 8), IA (*n* = 3), IC1 (*n* = 2), ID (*n* = 2), I-derived (*n* = 2), and IC2 (*n* = 1) (Suppl. material 1: table S5). Six insertion sites—mL2450, mL965, *cox1*P731, mL812, *cob*P393, and *cox2*P228—were highly prevalent, occurring in 71, 61, 53, 51, 36, and 31 mitotypes, respectively (Fig. 3B, Suppl. material 1: table S5). By contrast, five other sites (*atp9*P181, *cob*P823, *cox1*P709, *cox1*P1057, and *cox3*P333) were each found in only a single mitotype.

Each of these introns harbored 1–2 intronic ORFs (Suppl. material 1: table S5). They might encode ribosomal protein S3 (Rps3, in mL2450), GIY-YIG homing endonucleases (in mL812, mL965, *atp9*P181, *cox2*P228, *cob*P393, *cox1*P1057, and *nad1*P636), LAGLIDADG homing endonucleases (in mL1924, *nad5*P417, *cob*P490, *cob*P823, *cox1*P281, *cox1*P709, *cox1*P731, *cox3*P219, *cox3*P333, and *cox3*P631), or hypothetical proteins (in *cox1*P281) (Fig. 3C). Among all identified intron loci, an *rnl* intron (mL2450) encoding Rps3 was present in every mitotype, a characteristic feature of *Hypocreales* mitogenomes (Ren et al. 2021).

Although each sample possessed 2–12 intron insertion sites, the four-intron architecture (18.3%, 13/71) was the most common configuration, followed by three- and five-intron variants (15.5% each, *n* = 11) (Suppl. material 1: table S5). Other intron counts (fewer than three or more than five) were rare, each represented by no more than six mitotypes. Notably, intron number did not correlate simply with mitogenome size. For example, the two Russian strains (ZYJ0537 and ZYJ0541) harbored the maximum of 12 introns yet possessed relatively small mitogenomes (37.4–37.7 kb), whereas the two largest mitogenomes (ZYJ1057 and ZYJ1074; 39.3–40.3 kb) contained only 9–10 introns. The two most frequently detected mitotypes (1 and 2) contained six and two introns, respectively.

Based on the presence or absence patterns across all 18 intron loci, 49 distinct intron distribution profiles (designated profiles 1–49) were identified across the 71 mitotypes (Fig. 3D). Among these profiles, 13 were each shared by 2–4 mitotypes, and the remaining 36 were unique to single mitotypes. Notably, mitotypes with identical intron numbers could exhibit different intron distribution profiles. Further analysis of each intron locus revealed 16 distinct intron distribution patterns (designated patterns A–P) across all mitotypes (Fig. 3E). While 14 loci each displayed a unique pattern (A–M and O), two pairs of loci shared identical patterns: *cob*P823 and *atp9*P181 shared pattern N and were exclusive to strain ZYJ1074, whereas *cox3*P333 and *cox1*P1057 shared pattern P and were unique to strain ZYJ0189.

Comparative analysis of exonic sequences adjacent to intron insertion sites revealed co-conversion signatures upstream of *cob*P393, *cox1*P731, *cox2*P228, and *cox3*P631 (Suppl. material 2: fig. S6). Similar trends were observed for the rare *atp9*P181 (unique to ZYJ1074) and *cox1*P709 (exclusive to ZYJ1057) insertions; however, their single-strain occurrence prevented robust evaluation of co-conversion dynamics because of insufficient sampling. This co-conversion pattern was not detected in exonic sequences adjacent to other intron–exon junctions. Notably, nucleotide alterations were primarily localized within 20 bp of the insertion sites. Intriguingly, apart from an A/C transversion observed downstream of *atp9*P181, all other co-conversion events exclusively involved C/T or A/G transitions. The predominance of transitions (C/T or A/G) over transversions (A/C) in co-conversion events reflects the synergistic interplay between inherent molecular mechanisms and natural selection.

### Origin and evolution of introns in *C. militaris* mitogenomes

To investigate the evolutionary origin of the 18 introns in the *C. militaris* mitogenomes, potential intron mobility was assessed through (1) pairwise nucleotide sequence alignment of introns (including intronic ORFs) and (2) amino acid sequence comparisons of intron-encoded proteins (IEPs) using EMBOSS Stretcher. Global alignments revealed low to moderate nucleotide sequence identity (29.7–64.8%) among the introns (Suppl. material 1: table S6). Notably, mL812 and mL965 shared the highest nucleotide-level similarity (64.8%, coverage 90.1%), yet their corresponding IEPs shared only 42.2% amino acid identity (coverage 94.5%; Suppl. material 1: table S7). No significant sequence homology was observed across all intron pairs, effectively excluding intron jumping as a likely mechanism for their dispersal.

Online BLAST search results provided compelling evidence supporting horizontal gene transfer (HGT) in intron acquisition. The majority of the introns (e.g., mL812, *atp9*P181, *nad5*P417, *cob*P393, *cox1*P281, *nad1*P636, and *cox3*P219) showed the highest sequence similarity to introns from *Hypocreales*, particularly species within *Cordyceps*, *Beauveria*, and *Epichloe* (Suppl. material 1: table S8). Three introns—*cox2*P228, *cob*P490, and *cox1*P731—also showed maximal similarity to *Hypocreales* but were additionally similar to non-*Hypocreales* species. Collectively, these findings strongly suggest that horizontal gene transfer, particularly among closely related *Hypocreales* taxa, has played a significant role in the acquisition and diversification of introns in *C. militaris*.

Ancestral state reconstruction uncovered complex evolutionary trajectories for these introns (Suppl. material 2: fig. S7), with the notable exception of the universally conserved mL2450. Three principal evolutionary patterns were identified: (1) isolated single-gain events (e.g., *atp9*P181, *cob*P823, *cox3*P333, and *cox1*P1057); (2) extensive loss events (e.g., mL812 and *cox1*P731 experienced ≥ 12 independent losses each); and (3) complex gain–loss dynamics (e.g., *cox1*P281 showed ≥ 3 gains and ≥ 4 losses; *nad5*P417 exhibited a surprising 7:1 gain–loss ratio). Overall, loss events significantly outnumbered gain events across all loci (77 vs. 51, an ~3:2 ratio), indicating that intron loss has substantially outweighed intron gain during the evolution of *C. militaris*.

### Nucleotide variations among *C. militaris* mitogenomes

Sequence comparison of the 71 *C. militaris* mitotypes generated a multiple-sequence alignment of 54,478 bp. A total of 4,651 polymorphic sites were revealed, including 1,539 from genic regions (exonic and intronic combined) and 3,112 from intergenic regions (Suppl. material 1: table S9). Within the genic regions, exons and introns contributed 768 and 771 polymorphic sites, respectively. A clear divergence in the type of polymorphism was observed: single-nucleotide polymorphisms (SNPs) were the predominant variants in genic regions, while insertions/deletions (indels) were more common in intergenic regions. This pattern was especially pronounced in the distribution of indels, with intergenic regions harboring approximately six times more indel sites than genic regions (3,112 vs. 527). Based on the aligned sequences, SNP frequency was marginally greater in exonic regions (2.26%) than in intronic regions (1.91%), whereas intergenic regions exhibited a substantially higher SNP frequency (6.63%). At the whole-mitogenome level, the overall SNP frequency for *C. militaris* was calculated to be 2.48%.

To further characterize sequence evolution, the K2P distances and *K*a and *K*s substitution rates were calculated across the 71 *C. militaris* mitotypes. Among the 15 examined PCGs (14 core PCGs plus *rps3*), *cob* exhibited the largest K2P genetic distance, followed by *nad5* and *cox3*, indicating accelerated sequence divergence in these genes. In contrast, *atp8* showed the smallest K2P distance, reflecting its high conservation. Selective pressures were assessed using the *K*a/*K*s ratio, with all PCGs displaying values below 1, consistent with pervasive purifying selection. Gene-level variation in evolutionary dynamics was evident: *rps3* had the highest *K*a values, with *nad4* and *cox1* also showing elevated values (Suppl. material 2: fig. S8). Notably, *atp9* and *nad4L* had *K*a values of zero despite nonzero *K*s values, indicating that codon changes in these genes were exclusively synonymous—a pattern indicative of strong functional constraints. The *K*s values also varied substantially, with *cob* showing the highest value and *atp8* the lowest. Strikingly, *atp8* was entirely invariant across all mitotypes, with the K2P distance, *K*a, and *K*s all equal to zero, demonstrating absolute sequence conservation throughout the evolution of *C. militaris*.

### NUMTs in *C. militaris*

A comparative BLASTN analysis of the *C. militaris* mitogenome against its corresponding nuclear genome revealed considerable interstrain variability in the content of NUMTs, with the number of insertions per strain ranging from one to 34. The cumulative lengths of these fragments also varied widely, ranging from 66 bp to over 5.5 kb, suggesting a diverse array of mtDNA transfer events (Suppl. material 1: table S10). Notably, 44% of the examined strains contained only short NUMTs, characterized by single fragments shorter than 85 bp and total lengths under 332 bp, with no evidence of extensive mtDNA transfer. Of particular interest, strain ZYJ0778 harbored a particularly long NUMT of over 1,300 bp, providing compelling evidence for recent mtDNA transfer to the nuclear genome. Crucially, PCR validation, together with significantly lower read counts than those of the mitogenome, substantiated the authenticity of the NUMT (Suppl. material 2: fig. S9A, B), effectively excluding the possibility of sequencing artifacts or contamination.

### Divergent evolution between mitochondrial and nuclear genomes in *C. militaris*

**Figure 4.**
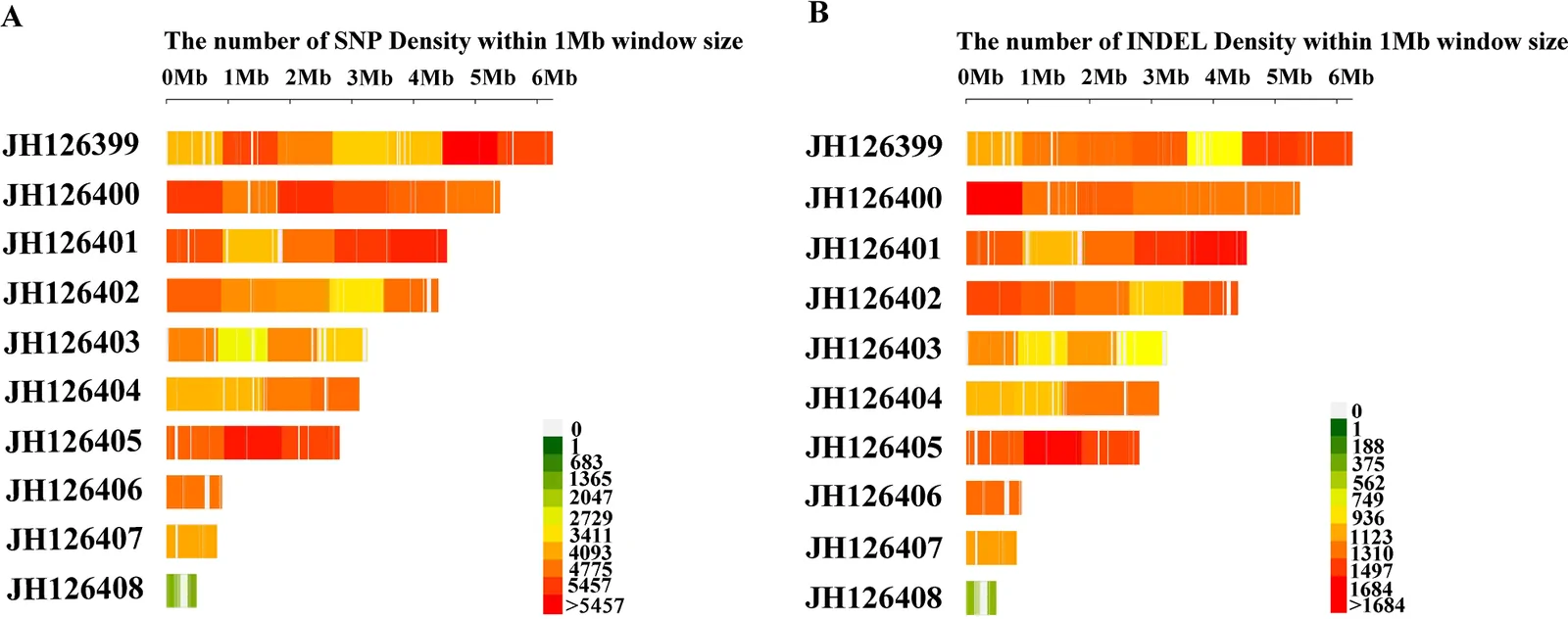
Distribution and density of SNPs and indels across 90 *C. militaris* strains. Variants were called using the nuclear genome of strain CM01 as the reference. Scaffold identifiers are shown on the left, and genomic positions are indicated along the x-axis in megabases (Mb). SNPs (**A**) and indels (**B**) were quantified in consecutive 1-Mb windows across each scaffold. Each colored block represents a single window, with the color scale indicating the number of SNPs or indels detected per window, ranging from low (green) to high (red) SNP or indel density.

To directly assess the evolutionary rate of the nuclear genome, a comprehensive analysis of SNPs and indels across the *C. militaris* nuclear genomes was performed. A total of 182,967 SNPs were identified among the 90 *C. militaris* strains, representing 0.57% of the genomic sites analyzed (Fig. 4). Most of these SNPs (66.83%) were located within protein-coding genes; of these, 78.04% were synonymous substitutions and 21.96% were nonsynonymous (Suppl. material 2: fig. S10). The transition-to-transversion (Ti/Tv) ratio varied substantially across samples (ranging from 0.25 to 3.07), suggesting differing levels of evolutionary constraint among genomic regions (Suppl. material 1: table S11). In contrast to the high abundance of SNPs, indels were far less frequent but exhibited a comparable genomic distribution. Most indels (60.39%) were located within upstream/downstream flanking regions and intergenic sequences (Suppl. material 2: fig. S11). Within coding regions, indels predominantly resulted in frameshift mutations (10.60%), whereas only 2.83% caused non-frameshift alterations (Suppl. material 1: table S12). Taken together, these results reveal distinct mutational spectra between SNPs and indels: coding regions tend to accumulate silent nucleotide changes, whereas indels are enriched in regulatory and noncoding sequences, reflecting different selective pressures and mutational mechanisms.

In stark contrast to the nuclear genome, the mitogenome of *C. militaris* exhibited a notably distinct and accelerated evolutionary trajectory. Alignment of mitogenomes from the same set of 90 strains yielded 54,485 aligned sites, comprising 469 singleton variable sites and 394 parsimony-informative sites, corresponding to an overall SNP frequency of 1.58%. Comparative analysis confirmed that mitogenomes evolve more rapidly than nuclear genomes, with SNP frequencies of 1.58% and 0.57%, respectively.

**Figure 5.**
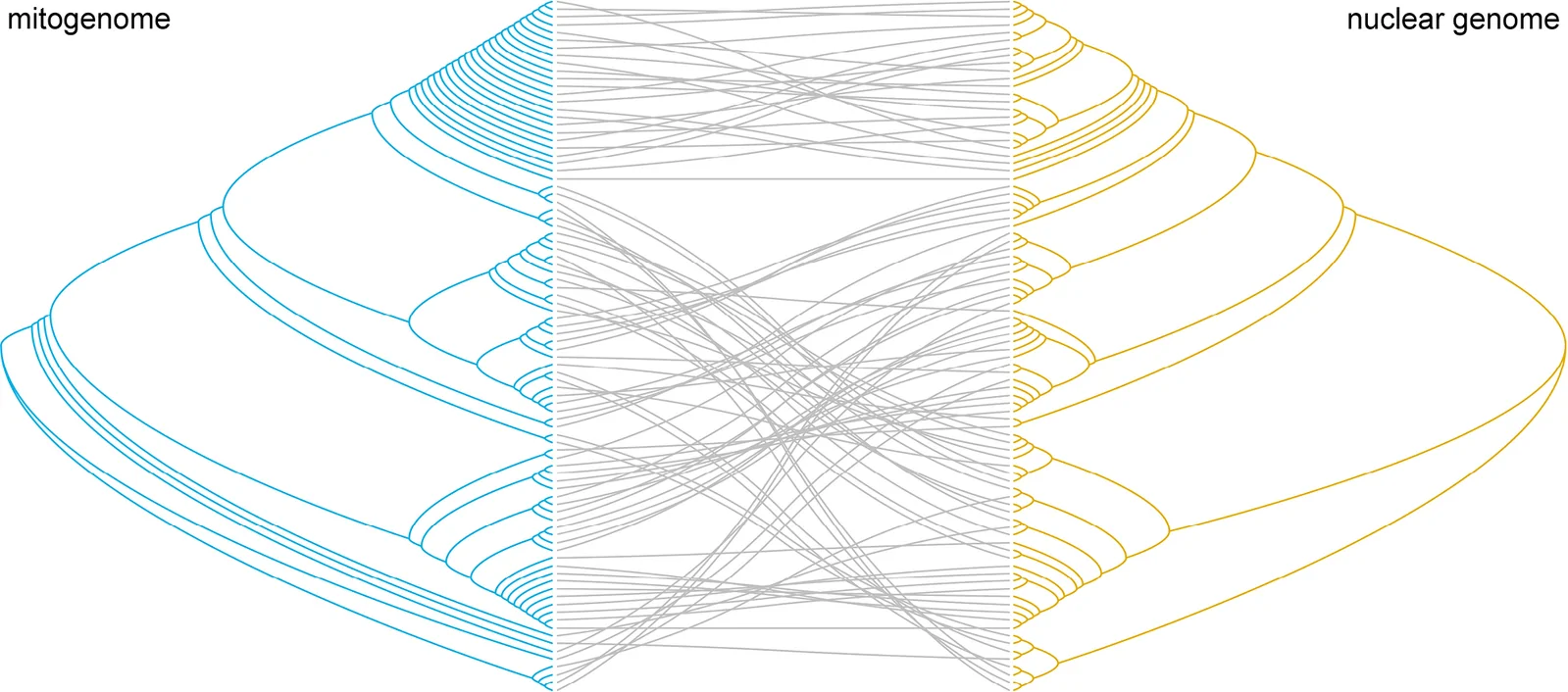
Comparison of phylogenetic topologies inferred from mitochondrial and nuclear genomes. The left and right trees were inferred from mitochondrial and nuclear datasets, respectively. Connecting lines link corresponding strains between the two phylogenies. Parallel connections indicate concordant topologies, whereas crossing lines reflect phylogenetic incongruence between the two genomic datasets. For clarity, strain labels are omitted from the figure; a fully annotated version is provided in Suppl. material 2: fig. S12.

Based on phylogenetic analyses of both mitochondrial and nuclear genomes, most strains exhibited largely congruent clustering patterns (Fig. 5; Suppl. material 2: fig. S12). However, topological discordances were detected for certain strains, indicating significant mitochondrial–nuclear discordance (SH test *P* < 0.0001; KH test *P* < 0.0001).

## Discussion

In this study, through a broad-scale comparison of 118 *C. militaris* mitogenomes from diverse origins, an expanded mitogenome size range (26.5–40.3 kb; Suppl. material 2: fig. S3A) is reported, exceeding prior records (26.5–33.9 kb) (Zhang et al. 2017a). In fungi, mitogenome expansion is typically driven by mobile intron proliferation and intergenic rearrangements (Tan et al. 2022; Zhong et al. 2022). Comparative studies across various *Cordyceps* species attribute mitogenome size variation to introns, intergenic regions, and ncORFs (Jia et al. 2025). In this dataset, intron number and total intron sequence length were the key drivers of mitogenome size variation in *C. militaris* (*R* = 0.948–0.969; Suppl. material 2: fig. S4A, B), whereas intergenic length showed only a weak correlation (*R* = 0.049; Suppl. material 2: fig. S4C). This minor role aligns with the compact architecture of *C. militaris* mitogenomes, wherein intergenic regions constitute only approximately 12.75% of the total length (Suppl. material 2: fig. S3B). Nevertheless, it is important to recognize that the geographic sampling in this study is uneven, with a strong representation of Chinese samples and comparatively sparse inclusion of samples from other countries. This sampling bias may compromise the generalizability of the diversity estimates and phylogenetic inferences, potentially underestimating the true intraspecific genetic diversity of *C. militaris* on a global scale. To comprehensively elucidate the species’ evolutionary history and population structure, future research should aim to incorporate a more extensive and geographically balanced sampling strategy encompassing the full natural distribution of the species.

Despite considerable variation in mitogenome size, the gene repertoire and structural order were highly conserved across all examined *C. militaris* mitogenomes. Although AT-skew varied among samples, GC-skew remained consistently positive across all mitogenomes. Similar nucleotide compositional patterns have also been reported in other entomopathogenic fungi, including *Cordyceps* (Jia et al. 2025) and *Moelleriella* (Xiong et al. 2025). These findings indicate that the weak AT-skew observed in *C. militaris* is consistent with the nucleotide compositional patterns commonly reported in fungal mitogenomes. A notable exception is the *trnG_1*(*acc*) gene, which was absent in 5% of the samples. Given that tRNA mutations are related to the efficiency of protein synthesis and may ultimately affect phenotypes (Lin et al. 2021), the diversity of *trnG* evolutionary patterns likely reflects adaptations within *C. militaris* and merits further investigation to elucidate its functional and evolutionary implications.

Mitogenome comparisons across different *Cordyceps* species have revealed significant variation in ncORF numbers, ranging from four to 21 (Jia et al. 2025). Similarly, the intraspecific survey of *C. militaris* found 0–4 ncORFs per mitogenome in intergenic regions, with a total of 10 ncORFs identified across all samples. Notably, contrary to earlier reports suggesting that ncORFs encode only uncharacterized proteins (Zhang et al. 2017a), the expanded dataset identified functional ncORFs encoding homing endonucleases. This finding updates the previous view by revealing that the *C. militaris* mitogenome harbors active mobile elements, suggesting that its intergenic diversity and functional complexity have been underestimated.

The comparative mitogenomic analysis of 118 *C. militaris* samples identified 71 distinct mitotypes, with a markedly skewed distribution. The two most frequently detected mitotypes (1 and 2) alone accounted for 33% of the samples, whereas 60 mitotypes were each represented by a single sample. This skewed distribution most likely reflects the combined effects of cultivated strain dissemination, germplasm exchange, and uneven geographic sampling, rather than natural population processes, given the composition of the dataset. Haplotype network analysis further revealed a complex, radially divergent topology, in which samples from fruiting bodies and strains were thoroughly intermixed, indicating extensive gene flow and an absence of reproductive isolation. Notably, despite the observed high haplotype diversity and complex population structure, syntenic analyses showed no indication of frequent rearrangements among the mitotypes (Suppl. material 2: fig. S5). This structural conservation at the mitogenomic level aligns with the considerable synteny reported across the genus *Cordyceps* (Jia et al. 2025).

In eukaryotes, mitochondrial introns often act as selfish genetic elements capable of self-splicing, contributing to their frequent gain and loss (Saldanha et al. 1993). Consistent with this dynamic, this study identified 18 intron insertion loci in *C. militaris* mitogenomes, with 2–12 introns per sample. This represents a significant increase from the eight loci previously known from five genes (Zhang et al. 2015). The expansion is attributable to the discovery of introns in new host genes (e.g., *atp9*, *nad1*, and *nad5*) and novel insertion sites within known intron-harboring genes (e.g., *cob*P823, *cox1*P709, *cox1*P1057, and *cox3*P333). Such intron dynamics are increasingly documented in fungi, with studies in *Boletus* (Li et al. 2021), *Agaricus bisporus* (Zhang et al. 2023), and *Thelephora ganbajun* (Li et al. 2024), all revealing substantial strain-specific variation in intron number and length.

Intron proliferation can occur via homing mechanisms (Megarioti and Kouvelis 2020) or through HGT across species and kingdoms (Husnik and McCutcheon 2018). In *C. militaris*, systematic BLAST analyses indicated that its introns originated from closely related homologs (Suppl. material 1: table S8). Nevertheless, intron loss appeared to dominate its evolutionary history (Suppl. material 2: fig. S7), unlike in *Tremella fuciformis*, in which no bias toward loss over gain was observed (Deng et al. 2020). These intron insertion sites exhibit frequent gain–loss dynamics, which often mediate the co-conversion of flanking exon regions (Torriani et al. 2014). Although a previous study detected such co-conversion in upstream exons of *cob*, *cox1*, *cox2*, and *cox3* in certain *C. militaris* strains (Zhang et al. 2015), the present study corroborates these earlier findings and additionally identifies co-conversion-like sequences upstream and downstream of *atp9*P181 and upstream of *cox1*P709. Notably, *atp9*P181 and *cox1*P709 are each unique to a single strain (ZYJ1074 and ZYJ1057, respectively). Thus, these events should be considered provisional until confirmed in a broader sample set. The observed variability in co-conversion patterns suggests possible mechanistic divergence in *C. militaris* intron homing. A more plausible explanation for this variation may involve the introduction of a conversion template through HGT or introgression (Repar and Warnecke 2017).

The evolutionary trajectory of mitochondrial genes is shaped by multiple forces, including mutation rate, purifying selection, and directional selection. Although synteny is largely conserved, *C. militaris* mitogenomes exhibit substantial size variation across their functional compartments: exonic regions (19,678–22,783 bp), intronic regions (3,020–14,327 bp), and intergenic regions (3,530–4,667 bp). At the sequence level, exons show moderately higher variability than introns—a pattern resembling that of *Thelephora ganbajun* (Li et al. 2024) but contrasting with that of *Beauveria bassiana* (Zhang et al. 2020). Overall, the mitogenome-wide SNP frequency in *C. militaris* (2.48%) is notably higher than those in related fungi, such as *Beauveria bassiana* (1.24%) (Peng et al. 2010) and *Isaria cicadae* (0.1%) (Fan et al. 2019), highlighting its elevated genomic plasticity. Despite this variability, analyses of K2P distances and substitution rates (*K*a and *K*s) reveal that the protein products of most core PCGs are highly conserved across the 71 mitotypes (Suppl. material 2: fig. S8). Together, these findings underscore the strong purifying selection acting on essential mitochondrial genes, which fundamentally underpins the organism’s evolutionary fitness.

The transfer of organellar DNA to the nucleus is a fundamental process in eukaryotic genome evolution, producing NUMTs that vary widely in number and length across species (Timmis et al. 2004). Documented fungal examples include a single 652 bp NUMT in *Tolypocladium inflatum* (Zhang et al. 2017b); four NUMTs longer than 388 bp in *Glarea lozoyensis* (Zhang et al. 2017c); and a 3,174 bp NUMT in *Fusarium graminearum* (Brankovics et al. 2018). A previous study of *C. militaris* strain V40-5 identified only five short NUMTs (totaling 278 bp) (Zhang et al. 2019), suggesting minimal transfer. However, such low detection rates may reflect methodological constraints, particularly reliance on public reference genomes rather than alignment against the corresponding nuclear genome of each individual. To overcome this limitation, large-scale BLASTN alignments of each *C. militaris* mitogenome against its corresponding nuclear genome were performed. The analysis revealed substantially greater transfer activity, with 1–34 NUMTs per strain and cumulative fragment lengths of up to 5.5 kb. Although the possibility of older NUMT degradation or technical artifacts (e.g., incomplete assembly) cannot be excluded, the authenticity of a long NUMT fragment in strain ZYJ0778 was confirmed by PCR validation and by its significantly lower sequencing depth relative to the mitogenome. This provides direct evidence of ongoing mitochondrial-to-nuclear DNA transfer in *C. militaris*.

Mitochondrial and nuclear genomes are maintained and inherited through distinct mechanisms, which can drive divergent evolutionary trajectories. Previous work comparing seven nuDNA and 14 mtDNA loci in *C. militaris* reported moderately faster nuDNA variation (SNP frequency: 1.24% vs. 0.71%, a 1.7-fold difference) (Zhang et al. 2019). In contrast, the genome-scale comparison revealed the opposite pattern: mtDNA accumulated mutations approximately 2.8 times faster than nuDNA, with SNP frequencies of 1.58% and 0.57% (based on comparisons across 90 strains), respectively. This disparity primarily stems from differing analytical scales: prior conclusions were based on a limited set of loci, whereas the present assessment integrates variations across the entire genomes, including both coding and non-coding regions. Thus, evolutionary rate estimates can differ substantially depending on whether loci or whole genomes are analyzed. The mechanisms underlying the pronounced mitochondrial–nuclear evolutionary rate disparity and their phenotypic consequences require functional validation. It is important to note that a uniform SNP-calling strategy was not employed for both genomes in this study: for the nuclear genome, which comprises multiple scaffolds, a reference-based mapping approach was used, whereas for the mitogenome, characterized by a single, compact molecule, direct sequence alignment was performed. Future studies could benefit from applying nuclear genome variation analysis techniques to mitochondrial data to further elucidate the functional consequences and biological significance of mitochondrial genomic variation.

Phylogenomic reconstructions based on mitochondrial and nuclear genomes yielded largely congruent—although not identical—topologies, reflecting the complex interplay between these two genetic compartments. This discordance may result from past hybridization, incomplete lineage sorting, or differential selective pressures acting on the two genomes. Notably, phylogenetic clustering showed no association with geographic origin, suggesting that spatial isolation has played a limited role in shaping their evolution. This observation is consistent with findings in other fungi, such as *Flammulina filiformis* (no clear mitogenomic phylogeography) (Tan et al. 2022) and *Corynespora cassiicola* (phylogenetic groups correlate with host/toxin but not geography) (Ma et al. 2021). Collectively, these results suggest that geographic isolation is not the primary driver of phylogenetic differentiation in these fungi; instead, other forces—such as host adaptation, selection, or hybridization—appear to play more significant roles.

## Conclusion

This study presents the first large-scale, integrated mitochondrial and nuclear genomic analysis of *C. militaris*, establishing a foundational map of its intraspecific organellar diversity. The findings demonstrate that, although mitochondrial architecture is structurally conserved, its evolution is remarkably dynamic, driven by frequent intron turnover, recurrent mtDNA transfer to the nucleus, and a significantly accelerated rate of variation compared with that of the nuclear genome. Notably, no clear association was observed between phylogenetic clustering and geographic origin. However, uneven geographic sampling may have influenced this pattern, and broader sampling will be required to fully assess the relationship between phylogeny and geographic distribution in *C. militaris*. Collectively, these findings advance the understanding of mitogenome evolution in *C. militaris* and provide an important foundation for future investigations into fungal genome evolution and organelle–nuclear genome interactions.

## Additional information

### Artificial Intelligence (AI) use

The authors accept full responsibility for the content of the manuscript, including the disclosure of any use of AI.

No AI tools were used in the preparation of this manuscript.

### Adherence to national and international regulations

All the fungal strains used in this study have been legally obtained, respecting the Convention on Biological Diversity (Rio Convention).

### Data availability

The assembled mitogenome sequences have been submitted to GenBank (https://www.ncbi.nlm.nih.gov/nuccore/) under accession numbers OM203115–OM203117 and PZ302635–PZ302699.

## Supplementary materials

Supplementary material 1 Supplementary table Data type xls Explanation note **table S1**. Metadata for the 118 *C. militaris* samples analyzed in this study. **table S2**. Fungal taxa employed in the taxonomic confirmation of the *C. militaris* samples used in this study. **table S3**. GenBank accession numbers of the mitogenomes employed in the phylogenetic analysis. **table S4**. Comparison of mitochondrial gene order across the 71 *C. militaris* mitotypes. **table S5**. Characteristics of introns and intron-encoded ORFs identified in 71 *C. militaris* mitotypes. **table S6**. Pairwise global alignment of intron nucleotide sequences (EMBOSS Stretcher). **table S7**. Pairwise global alignment of intron-encoded protein sequences (EMBOSS Stretcher). **table S8**. BLASTN search results for *C. militaris* mitochondrial introns against the NCBI database. **table S9**. Sequence variations across exonic, intronic, and intergenic regions in 71 *C. militaris* mitotypes. **table S10**. BLASTN detection of NUMTs across 90 *C. militaris* strains. **table S11**. Catalog and genomic distribution of SNPs identified across 90 *C. militaris* nuclear genomes. **table S12**. Catalog and genomic distribution of indels identified across 90 *C. militaris* nuclear genomes. https://binary.pensoft.net/file/1743923This dataset is made available under the Open Database License (http://opendatacommons.org/licenses/odbl/1.0/). The Open Database License (ODbL) is a license agreement intended to allow users to freely share, modify, and use this Dataset while maintaining this same freedom for others, provided that the original source and author(s) are credited. Li-Yuan Ren, Shu Zhang, Yong-Jie Zhang

Supplementary material 2 Supplementary images Data type pdf Explanation note **figure S1**. Phylogenetic analysis for the taxonomic confirmation of 118 *C. militaris* samples. The tree was constructed from a concatenated alignment of six nuclear loci (ITS, LSU, SSU, *tef1α*, *rpb1*, and *rpb2*), with *Beauveria bassiana* and *B. staphylinidicola* as outgroups. Support values from ML (bootstrap ≥ 75%) and BI (posterior probability ≥ 0.75) are shown at the nodes. **figure S2**. PCR validation of *trnG_1* gene absence. The electrophoretogram confirms that six *C. militaris* samples lack the *trnG_1* gene. Strains ZYJ0692 and ZYJ0899 were included as positive controls. **figure S3**. Comparative mitogenome architecture of 118 *C. militaris* samples. (**A**) Length distributions of exonic, intronic, and intergenic regions. (**B**) Mean proportional composition of the three genomic compartments. **figure S4**. Correlations of mitogenome size with intron length (**A**), intron number (**B**), and intergenic region length (**C**). **figure S5**. Mitogenome collinearity across 71 *C. militaris* mitotypes. The alignment illustrates conserved syntenic blocks shared among mitogenomes, with lines connecting homologous regions. The reference gene order of strain FSXU0234G is shown above, with rRNAs in red, 14 core PCGs in yellow, and tRNAs in blue. **figure S6**. Co-conversion tracts in exonic sequences flanking intron insertion sites (**A–G**). For each intron locus, the chart displays the observed frequency of specific nucleotide combinations at consecutive exon positions adjacent to the intron–exon junction (5'→3' orientation). **figure S7**. Reconstruction of intron gain and loss events across 71 *C. militaris* mitotypes (**A–P**). Hollow white lines indicate intron absence (loss), while solid black lines denote intron presence (gain or retention). **figure S8**. Genetic distances and substitution rates of 15 PCGs across 71 *C. militaris* mitotypes. (**A**) Nonsynonymous substitution rate (*K*a). (**B**) Synonymous substitution rate (*K*s). (**C**) Ratio of nonsynonymous to synonymous substitutions (*K*a/*K*s). (**D**) Kimura two-parameter (K2P) genetic distance. **figure S9**. PCR verification and sequencing evidence for a NUMT. (**A**) Electrophoresis profiles confirm the presence of NUMTs in the ZYJ0778 nuclear genome that correspond to BLAST alignments. (**B**) Schematic diagram showing Illumina sequencing reads aligned to the mitogenome and NUMTs of ZYJ0778, with the two nuclear genes *rpb1* and *tef1α* acting as controls. **figure S10**. Distribution of SNPs across different nuclear genomic regions. **figure S11**. Distribution of indels across different nuclear genomic regions. **figure S12**. Comparison of phylogenetic topologies inferred from the mitochondrial and nuclear genomes, with sample names indicated. https://binary.pensoft.net/file/1743924This dataset is made available under the Open Database License (http://opendatacommons.org/licenses/odbl/1.0/). The Open Database License (ODbL) is a license agreement intended to allow users to freely share, modify, and use this Dataset while maintaining this same freedom for others, provided that the original source and author(s) are credited. Li-Yuan Ren, Shu Zhang, Yong-Jie Zhang

Supplementary material 3 Alignment used to construct the phylogenetic tree shown in Suppl. material 2: fig. S1 Data type phy https://binary.pensoft.net/file/1743925This dataset is made available under the Open Database License (http://opendatacommons.org/licenses/odbl/1.0/). The Open Database License (ODbL) is a license agreement intended to allow users to freely share, modify, and use this Dataset while maintaining this same freedom for others, provided that the original source and author(s) are credited. Li-Yuan Ren, Shu Zhang, Yong-Jie Zhang

Supplementary material 4 Alignment used to construct the phylogenetic tree shown in Suppl. material 2: fig. S7 Data type phy https://binary.pensoft.net/file/1743926This dataset is made available under the Open Database License (http://opendatacommons.org/licenses/odbl/1.0/). The Open Database License (ODbL) is a license agreement intended to allow users to freely share, modify, and use this Dataset while maintaining this same freedom for others, provided that the original source and author(s) are credited. Li-Yuan Ren, Shu Zhang, Yong-Jie Zhang

Supplementary material 5 Alignment used to construct the mitochondrial phylogenetic tree shown in Fig. 5 Data type phy https://binary.pensoft.net/file/1743927This dataset is made available under the Open Database License (http://opendatacommons.org/licenses/odbl/1.0/). The Open Database License (ODbL) is a license agreement intended to allow users to freely share, modify, and use this Dataset while maintaining this same freedom for others, provided that the original source and author(s) are credited. Li-Yuan Ren, Shu Zhang, Yong-Jie Zhang

Supplementary material 6 Alignment used to construct the nuclear phylogenetic tree shown in Fig. 5 Data type phy https://binary.pensoft.net/file/1743928This dataset is made available under the Open Database License (http://opendatacommons.org/licenses/odbl/1.0/). The Open Database License (ODbL) is a license agreement intended to allow users to freely share, modify, and use this Dataset while maintaining this same freedom for others, provided that the original source and author(s) are credited. Li-Yuan Ren, Shu Zhang, Yong-Jie Zhang
