# Supplementary material for: Evolutionary insights into Cordyceps militaris from mitogenome dynamics and genome-wide variation
Source: IMA Fungus. 2026 Aug 12;17:e195879. doi: 10.3897/imafungus.17.195879 (PMC13491011; doi:10.3897/imafungus.17.195879)
Supplement: Supplementary file 1 [file imafungus-17-e195879-s002.pdf]

Figure S1. Phylogenetic analysis for the taxonomic confirmation of 118 *C. militaris* samples. The tree was constructed from a concatenated alignment of six nuclear loci (ITS, LSU, SSU, *tef1α*, *rpb1*, and *rpb2*), with *Beauveria bassiana* and *B. staphylinidicola* as outgroups. Support values from ML (bootstrap ≥75%) and BI (posterior probability ≥0.75) are shown at the nodes.

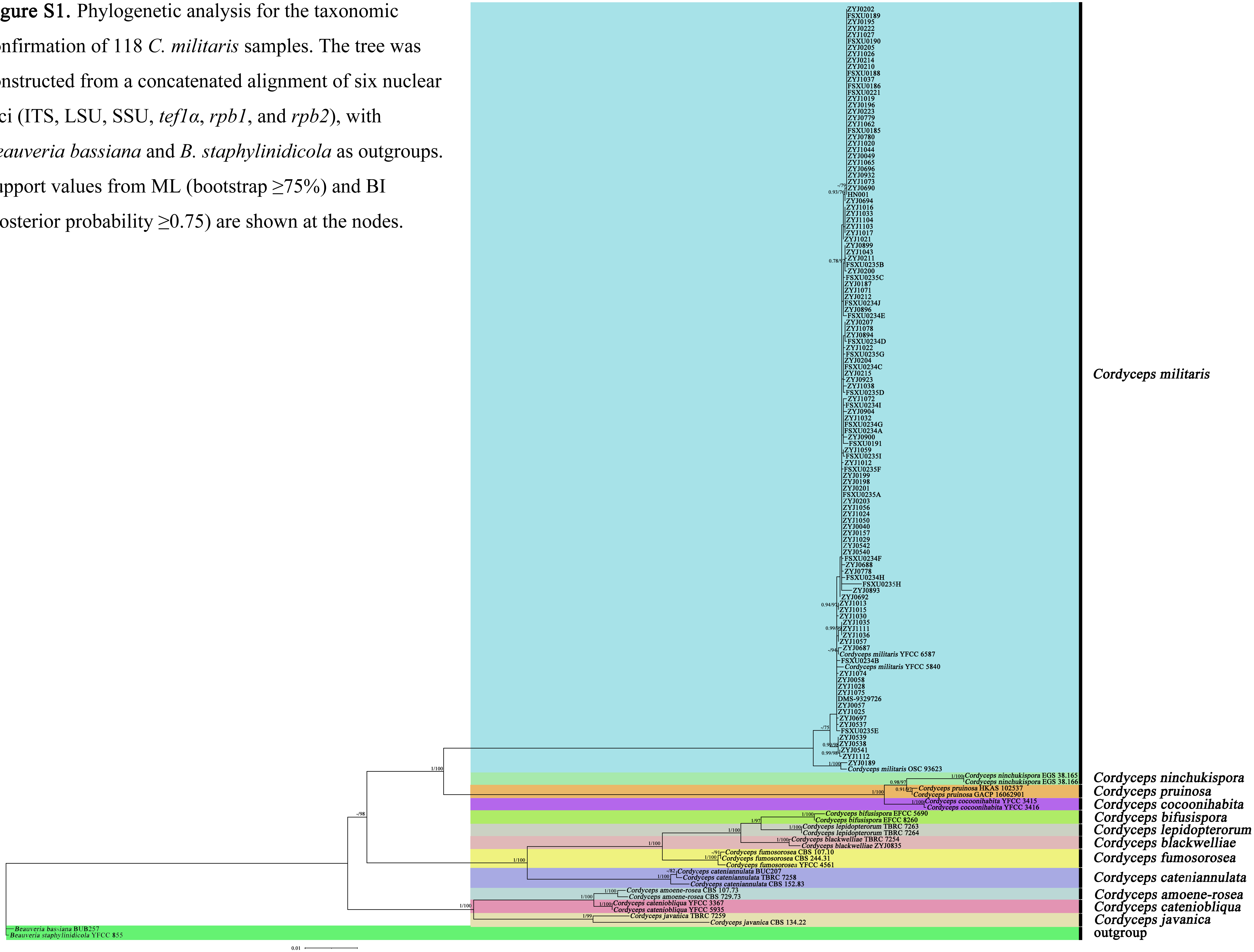

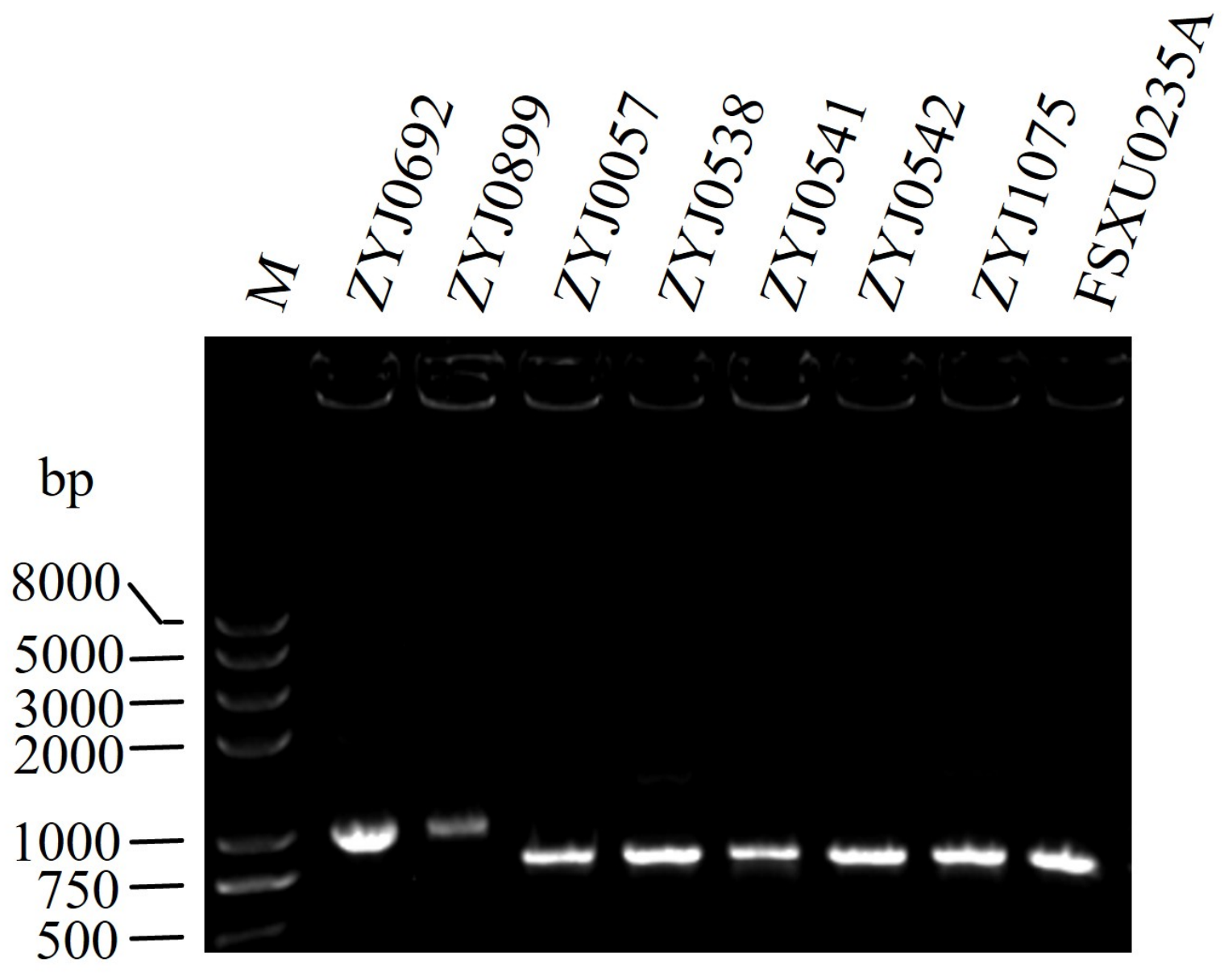

Figure S2. PCR validation of *trnG_1* gene absence. The electrophoretogram confirms that six *C. militaris* samples lack the *trnG_1* gene. Strains ZYJ0692 and ZYJ0899 were included as positive controls.

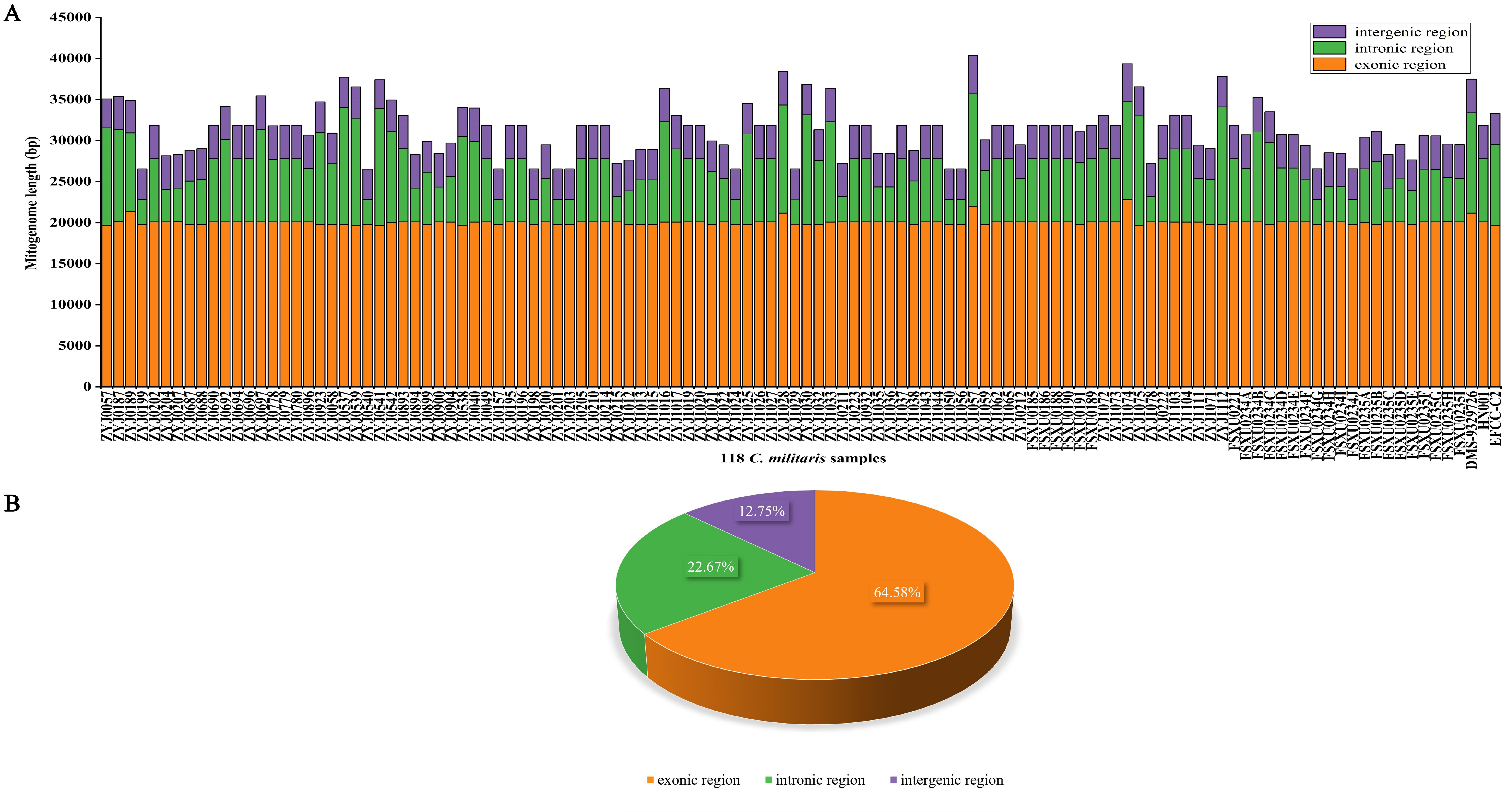

Figure S3. Comparative mitogenome architecture of 118 *C. militaris* samples. (**A**) Length distributions of exonic, intronic, and intergenic regions. (**B**) Mean proportional composition of the three genomic compartments.

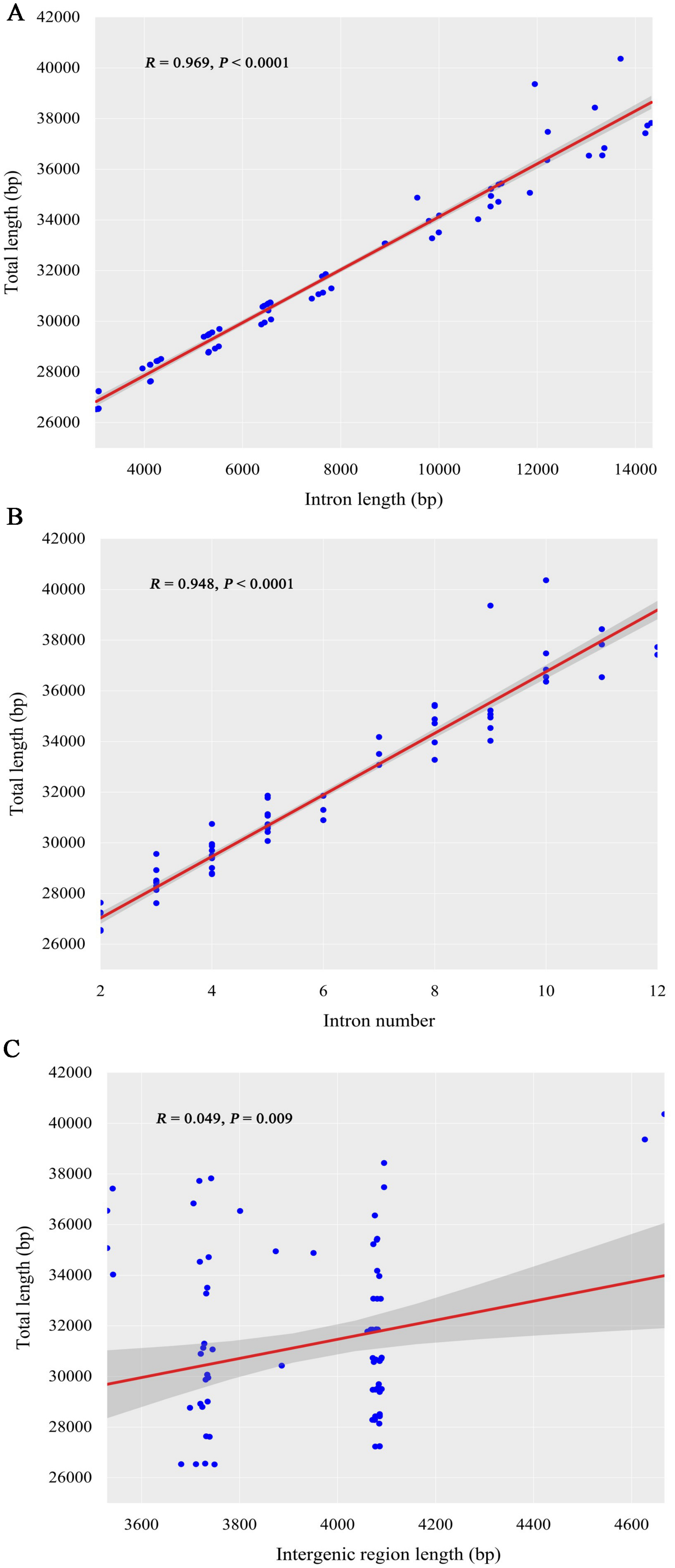

Figure S4. Correlations of mitogenome size with intron length (**A**), intron number (**B**), and intergenic region length (**C**).

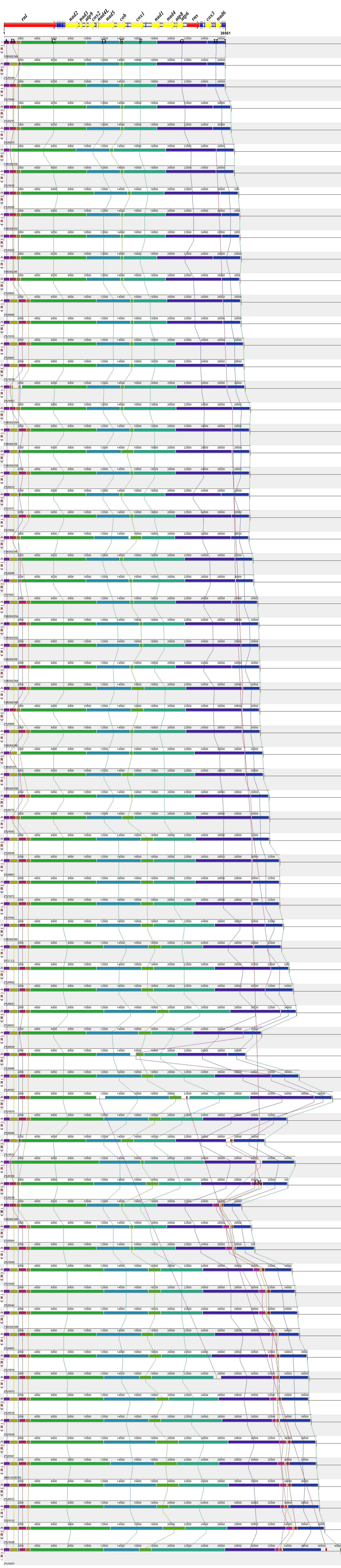

Figure S5. Mitogenome collinearity across 71 *C. militaris* mitotypes. The alignment illustrates conserved syntenic blocks shared among mitogenomes, with lines connecting homologous regions. The reference gene order of strain FSXU0234G is shown above, with rRNAs in red, 14 core PCGs in yellow, and tRNAs in blue.

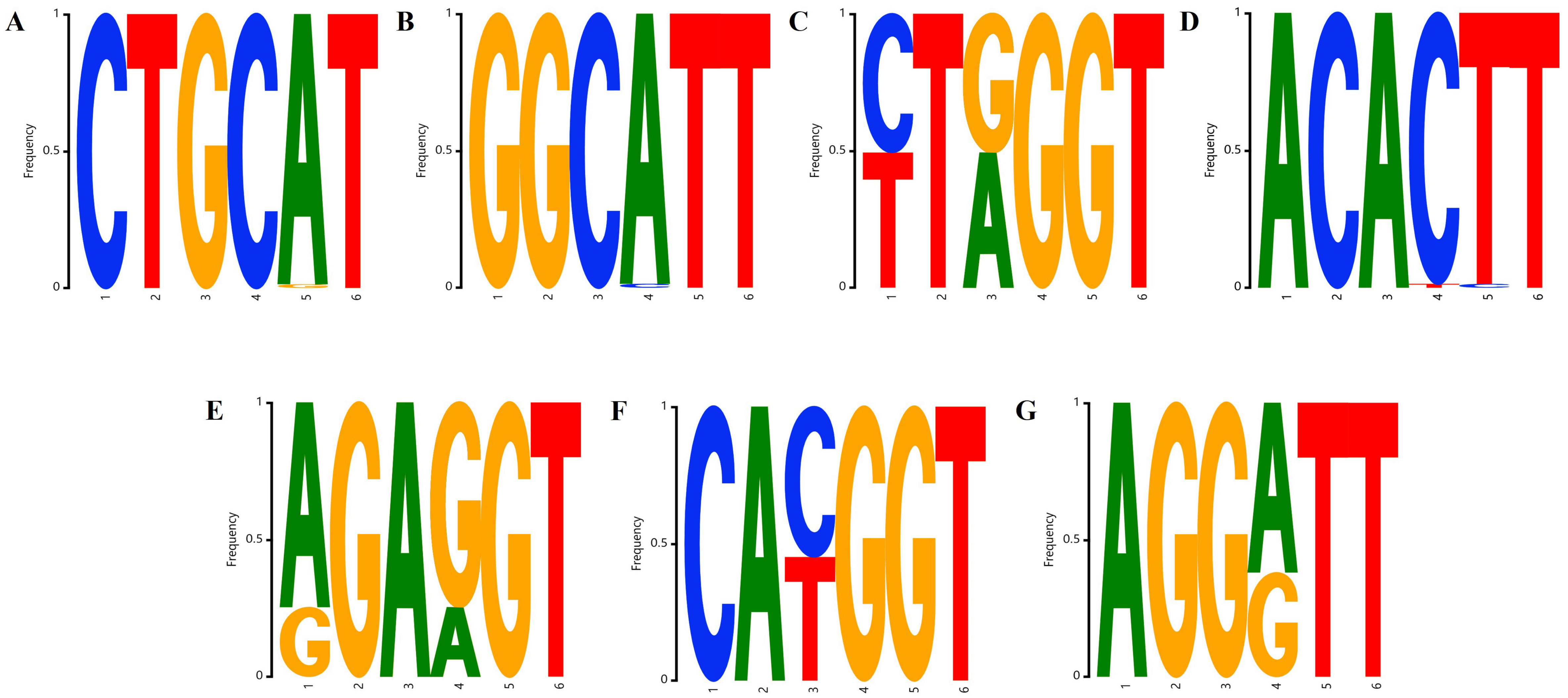

Figure S6. Co-conversion tracts in exonic sequences flanking intron insertion sites (A–G). For each intron locus, the chart displays the observed frequency of specific nucleotide combinations at consecutive exon positions adjacent to the intron–exon junction (5′→3′ orientation).

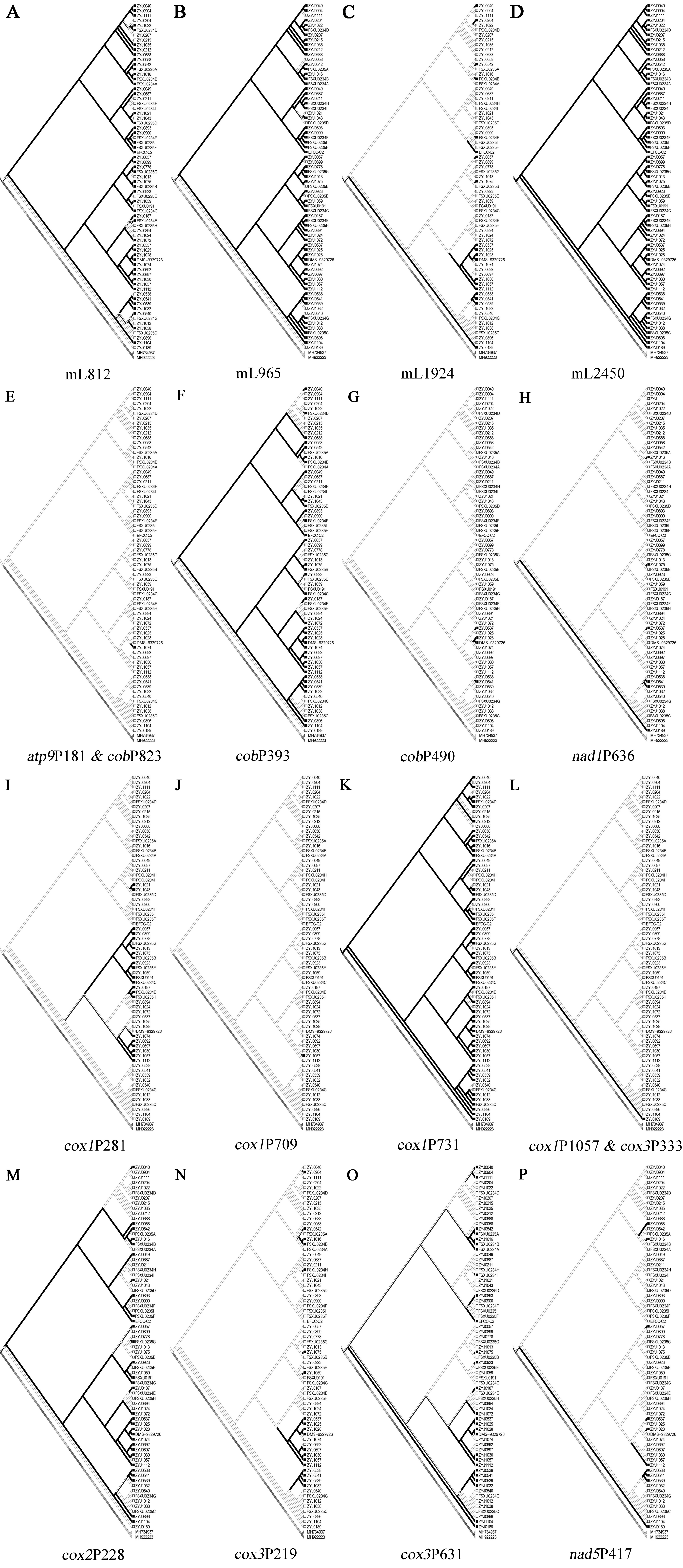

Figure S7. Reconstruction of intron gain and loss events across 71 *C. militaris* mitotypes (A–P) .

Hollow white lines indicate intron absence (loss), while solid black lines denote intron presence (gain or retention).

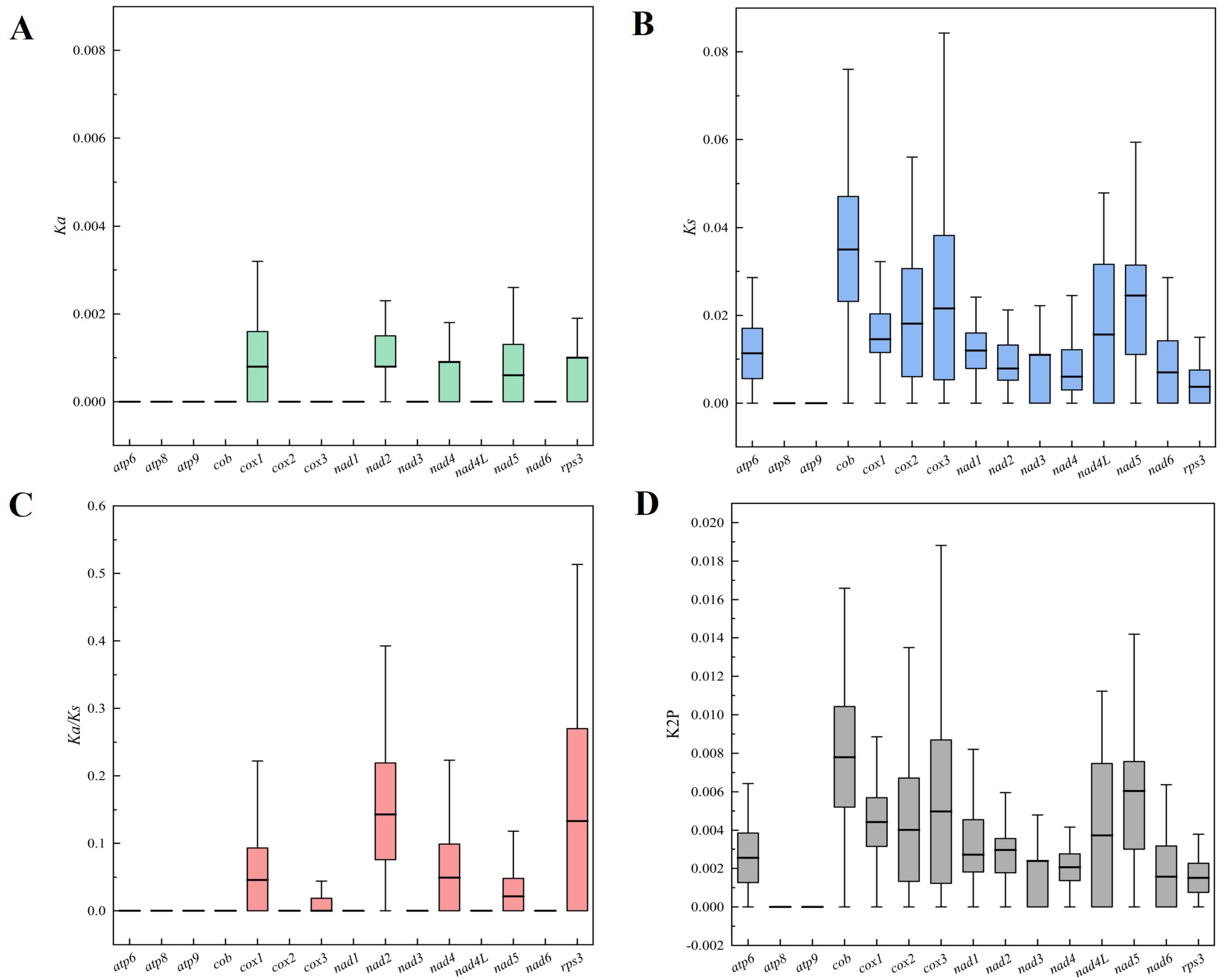

Figure S8. Genetic distances and substitution rates of 15 PCGs across 71 *C. militaris* mitotypes. (**A**) Nonsynonymous substitution rate (*Ka*). (**B**) Synonymous substitution rate (*Ks*). (**C**) Ratio of nonsynonymous to synonymous substitutions (*Ka*/*Ks*). (**D**) Kimura two-parameter (K2P) genetic distance.

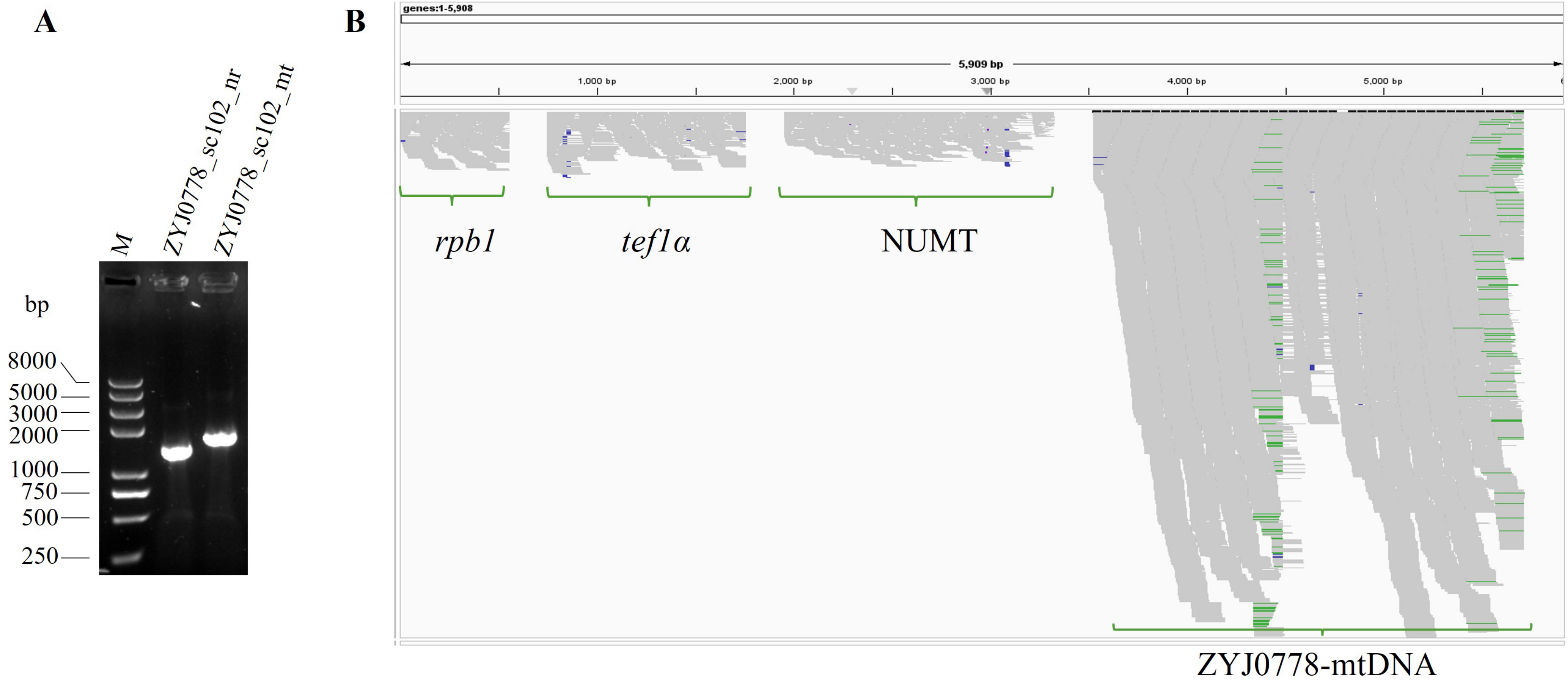

Figure S9. PCR verification and sequencing evidence for a NUMT. (**A**) Electrophoresis profiles confirm the presence of NUMTs in the ZYJ0778 nuclear genome that correspond to BLAST alignments. (**B**) Schematic diagram showing Illumina sequencing reads aligned to the mitogenome and NUMTs of ZYJ0778, with the two nuclear genes *rpb1* and *tef1α* acting as controls.

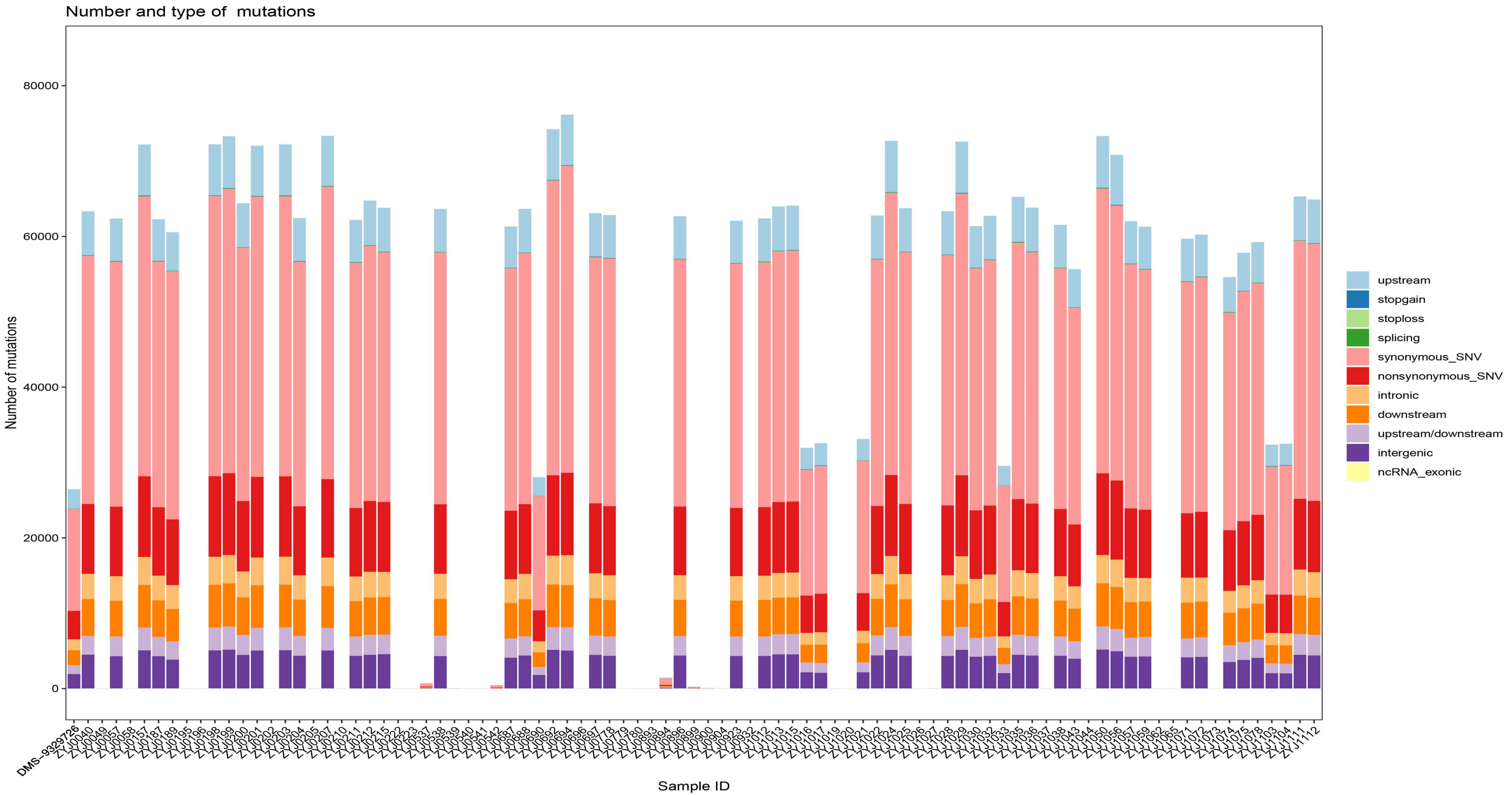

Figure S10. Distribution of SNPs across different nuclear genomic regions.

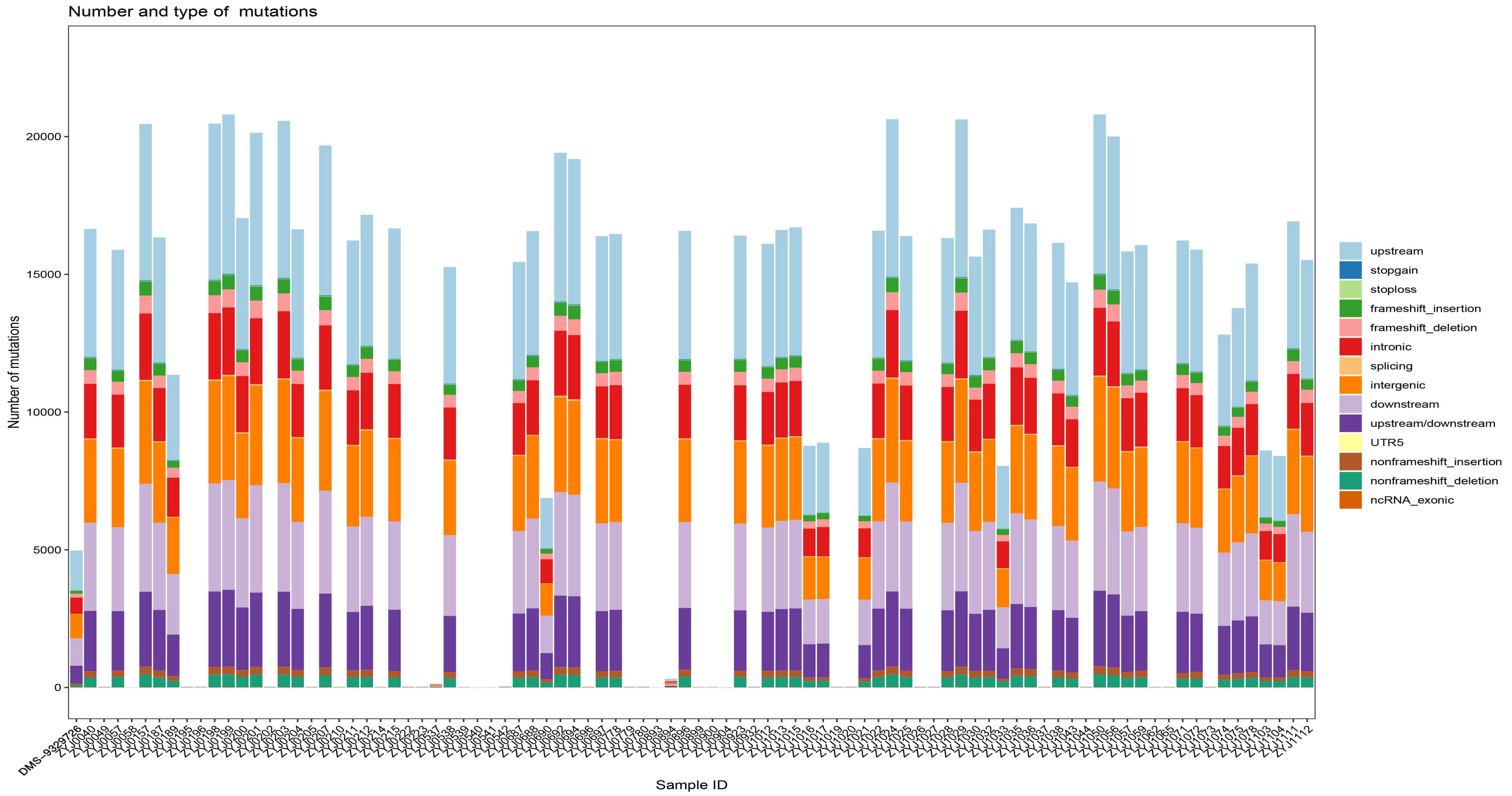

Figure S11. Distribution of indels across different nuclear genomic regions.

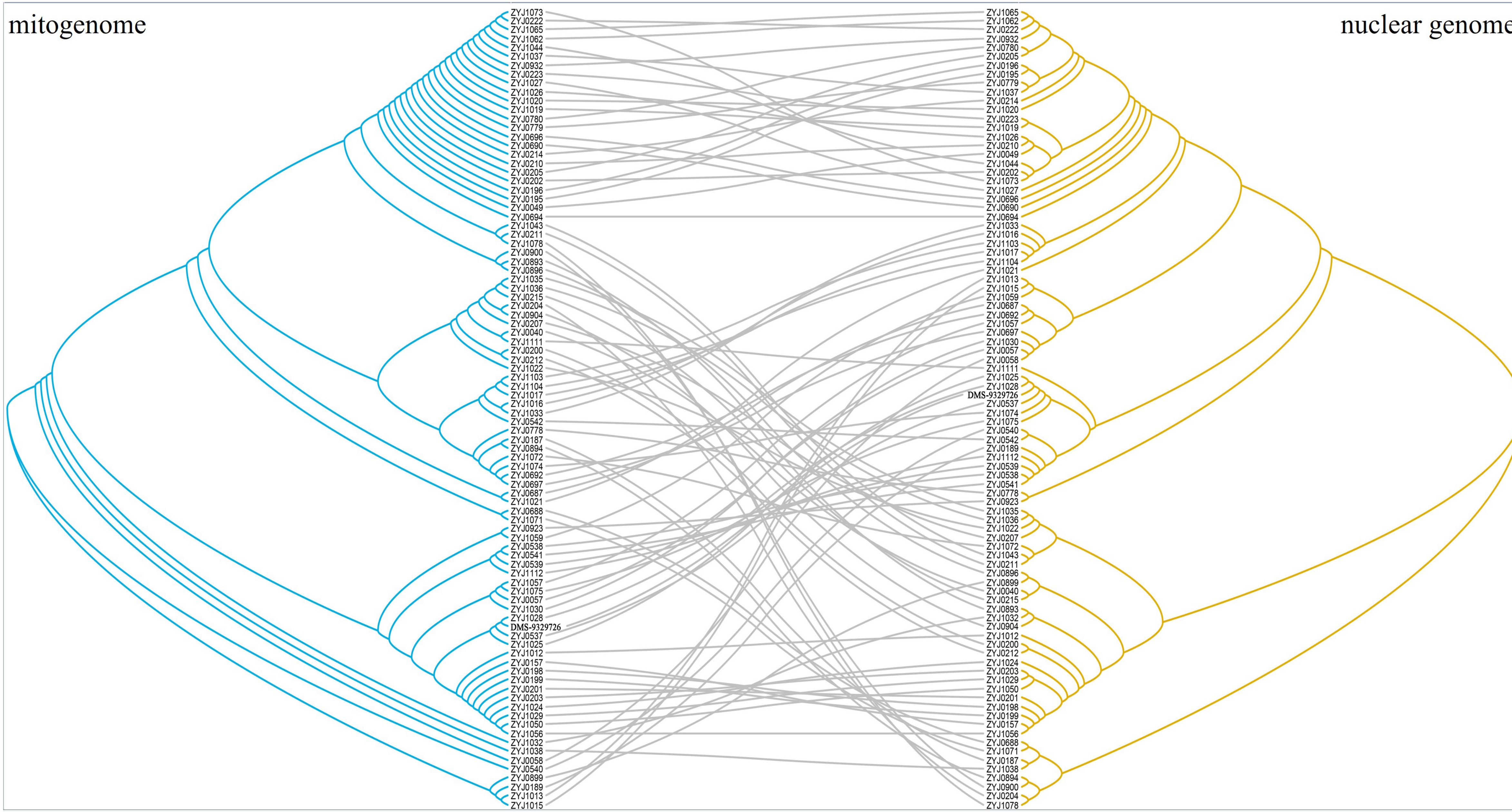

Figure S12. Comparison of phylogenetic topologies inferred from the mitochondrial and nuclear genomes, with sample names indicated.
